# Soil microbial community parameters affected by microplastics and other plastic residues

**DOI:** 10.3389/fmicb.2023.1258606

**Published:** 2023-10-12

**Authors:** Yüze Li, Yuting Hou, Quanming Hou, Mei Long, Ziting Wang, Matthias C. Rillig, Yuncheng Liao, Taiwen Yong

**Affiliations:** ^1^Sichuan Engineering Research Center for Crop Strip Intercropping System, College of Agronomy, Sichuan Agricultural University, Chengdu, China; ^2^College of Agronomy, Northwest A&F University, Xianyang, China; ^3^College of Agronomy, Guangxi University, Nanning, China; ^4^Guangxi Key Laboratory of Sugarcane Biology, Nanning, China; ^5^Freie Universität Berlin, Institute of Biology, Berlin, Germany; ^6^Berlin-Brandenburg Institute of Advanced Biodiversity Research (BBIB), Berlin, Germany

**Keywords:** plastics, dose–response, microorganism, microbial ecology, microbial functionality, environmental risk

## Abstract

**Introduction:**

The impact of plastics on terrestrial ecosystems is receiving increasing attention. Although of great importance to soil biogeochemical processes, how plastics influence soil microbes have yet to be systematically studied. The primary objectives of this study are to evaluate whether plastics lead to divergent responses of soil microbial community parameters, and explore the potential driving factors.

**Methods:**

We performed a meta-analysis of 710 paired observations from 48 published articles to quantify the impact of plastic on the diversity, biomass, and functionality of soil microbial communities.

**Results and discussion:**

This study indicated that plastics accelerated soil organic carbon loss (effect size = −0.05, *p* = 0.004) and increased microbial functionality (effect size = 0.04, *p* = 0.003), but also reduced microbial biomass (effect size = −0.07, *p* < 0.001) and the stability of co-occurrence networks. Polyethylene significantly reduced microbial richness (effect size = −0.07, *p* < 0.001) while polypropylene significantly increased it (effect size = 0.17, *p* < 0.001). Degradable plastics always had an insignificant effect on the microbial community. The effect of the plastic amount on microbial functionality followed the “hormetic dose–response” model, the infection point was about 40 g/kg. Approximately 3564.78 μm was the size of the plastic at which the response of microbial functionality changed from positive to negative. Changes in soil pH, soil organic carbon, and total nitrogen were significantly positively correlated with soil microbial functionality, biomass, and richness (*R*^2^ = 0.04–0.73, *p* < 0.05). The changes in microbial diversity were decoupled from microbial community structure and functionality. We emphasize the negative impacts of plastics on soil microbial communities such as microbial abundance, essential to reducing the risk of ecological surprise in terrestrial ecosystems. Our comprehensive assessment of plastics on soil microbial community parameters deepens the understanding of environmental impacts and ecological risks from this emerging pollution.

## 1. Introduction

The eco-functionality refers to the ability of the ecosystem to perform its functions and processes in a balanced and sustainable manner (Kremen, 2005). These functions include nutrient cycling, carbon sequestration, carbon mineralization, and overall maintenance of ecosystem health (Teague and Kreuter, 2020). Specifically, for, the microbes in the soil ecosystem engage in various above-mentioned processes (Bardgett and van der Putten, 2014; Zhou et al., 2020). Microbial biomass refers to the size or population of its community, and microbial α-diversity refers to the number of species in its community at the local scale, while β-diversity focuses on the regional scale. The microbial structure emphasizes the overall pattern of the community. In addition, microbial α-diversity is frequently found to be negatively correlated or decoupled from functionality under global change factors, which is different from the findings in plant and animal communities (Zhou et al., 2020).

Since the mass production of plastic products, plastic pollutants have been accumulating in soil, water, and even in the most remote environments in just a few decades (Barnes et al., 2009; Kane et al., 2020; Rillig and Lehmann, 2020). Plastic waste accumulation is an important environmental symbol of the Anthropocene, and plastic pollution is quickly becoming a serious global ecological and environmental problem (Brandon et al., 2019). Research on plastics initially focused on aquatic ecosystems such as lakes and oceans (Thompson et al., 2004), while as the main carrier of plastic, the content in terrestrial ecosystems is estimated to be 4–23 times higher than that in oceans (Horton et al., 2017). With several sources such as irrigation, sludge application, fertilization, and atmospheric deposition, soil systems become important sinks for plastics (Tian et al., 2022). Once incorporated into the soil, the migration of plastics can be driven by leaching, animal disturbance, and agricultural practices; they can be aged or transformed by light, oxygen, and microbes, eventually breaking down into smaller plastics like microplastics and even nanoplastics that enter soil microbes directly (Liu P. et al., 2021). Moreover, these plastics, with various size classes including macroplastics (> 25 mm), mesoplastics (> 5 mm and 25 mm), microplastics (> 1 μm and 5 mm), and nanoplastics (< 1 μm) (Hanvey et al., 2016), have a significant impact on soil physicochemical properties, microbes and the biogeochemical cycles mediated by them (De Souza Machado et al., 2018). The studies on the microecological effects of microplastics have been conducted (Wang et al., 2022a,b; Salam et al., 2023), but it is still unclear how all plastics affect soil microbes and their functionality.

Numerous studies indicate that plastics have a positive impact on soil microbial activity by serving as an external carbon source that microbes might utilize (Zhou et al., 2021; Wu et al., 2022). After being incorporated into the soil, plastics exert direct physical effects on the formation and stability of soil aggregates, or mediated by soil biota and organic matter (Wang et al., 2022a). Likewise, plastics can be viewed as a form of carbon input (Rillig et al., 2021); in this case, soil microbes will attach to the surface of plastics and create a unique ecological community called the “plastisphere” (Zhou et al., 2021). In addition, microbial enzymes break down the chemical bonds of plastic to produce breakdown products, which subsequently enter cells (Zhang X. et al., 2021). However, the long-term accumulation of plastics represents an input of recalcitrant carbon resources that are difficult for microbes to utilize as substrates (Guo et al., 2022; Wang et al., 2022c). As a result, the microbial diversity decreased due to the extinction of taxa that cannot adapt to the current environment (Xu C. et al., 2020). Other studies found owing to the major disruption produced by plastics, the direction and magnitude of changes in various soil microbial parameters are not always the same (Yuan et al., 2023), and insignificant impacts on microbes were even reported (Blocker et al., 2020). The responses of soil microbial communities to plastics have been thoroughly reviewed, and it has been found that they can vary based on the characteristics of the plastic, the microbial groups, and the soil qualities (Buks and Kaupenjohann, 2020; Mbachu et al., 2021; Wang J. et al., 2021; Zhang X. et al., 2021). Nevertheless, the relative influence of these factors on microbial parameters in various contexts has not been thoroughly investigated yet. In analogy to the addition of pyrogenic recalcitrant carbon substrates like biochar (Rillig et al., 2021), a related issue is whether (and if so, how) plastics alter the soil carbon, nitrogen, and phosphorus cycle.

Previously, focusing only on microplastics, a review summarized that the effects of microplastic properties on behaviors of larger organisms fit the hormetic dose–response model (Agathokleous et al., 2021a). The impacts of microplastics on individual organisms were attributed to numerous aspects like ingestion, oxidative stress, and reproductive toxicity (Wang et al., 2022d). Thus, a prevalent concept that appears to be largely inspired by investigations of these macroorganisms is whether microbes have a similar dose–response to plastics (Shi Z. et al., 2022). However, given that soil microbial communities are diverse and abundant, and the activities of microbes clearly differ from macroorganisms, thus requiring integration of results from multiple studies to determine whether the response of soil microbial parameters to plastics (with wider size class including microplastics) can be similar to or distinct from results of larger organisms (Amaral-Zettler et al., 2015). Hence, our understanding of how plastics affect soil microbes lags far behind that of larger organisms. These knowledge gaps swamp our predictions of plastics’ impact on microbial parameters, thereby constraining the rise of comprehensive and effective policies against plastic pollution.

Here, by using meta-analysis, for the first time we comprehensively evaluated the response of soil microbes to plastics, incorporating a large number of microbial indicators and context information of plastics. The focus is on the impact of plastics on soil microbial communities in terrestrial ecosystems. As microbes can be affected by plastic properties, exposure time, soil properties, and microbial groups that with distinct physiological and behavioral characteristics (Buks and Kaupenjohann, 2020; Mbachu et al., 2021). We hypothesized that different plastic types, sizes, amounts, incubation times, and changes in soil environmental properties would all have diverse effects and be important predictors of microbial parameters (H1). Drawing on the ecotoxicological effects of plastics on microbial diversity and stimulated basal respiration (Huo et al., 2022; Zhang J. et al., 2022), we also hypothesized that plastics would decrease microbial diversity but increase soil functionality. Thus, plastics could lead to a negative correlation or decoupling between microbial diversity and functionality (H2).

## 2. Materials and methods

### 2.1. Literature search and data collection

Peer-reviewed articles that report the effect of plastic residue on soil microbial community parameters, including microbial biomass, diversity, and functionality were searched by accessing the databases ISI Web of Knowledge and China Knowledge Resource Integrated Database from 1980 up to May 2021, with no temporal scale restriction. To collect the published data about the effect of plastics on soil microbial community parameters, the keyword combinations [(“microplastic*” OR “macroplastic*” OR “nanoplastic*” OR “plastic resid*”) AND (“microbial community” OR “microbe”) AND (“soil”)] were used as the first step to screen potentially related articles. Then the articles meeting the following criteria were selected for the dataset of our meta-analysis to minimize the nonindependence: (i) studies with experimental design quantifying the effect of plastic residue with a comparison between treatment with and without plastic material incorporated into soil; (ii) at least one soil microbial community parameter (biomass, diversity, or functionality) was reported in the article; (iii) the control and plastic amendment treatments had the same experimental conditions, i.e., the biome, soil type, and duration were similar between treatments; (iv) the various data were corresponding strictly on the spatiotemporal scale; (v) if the relevant data of more than one season/year were reported in a study, only the last data was extracted for our meta-analysis; (vi) the replication levels of both control and treatment were described. For studies with experimental design involved more than one manipulating factor, only the data from the control and the plastic treatment were extracted and included in the dataset, to avoid any potential interaction effects.

Apart from the soil microbial community parameters, if available, we also extracted information about soil texture, soil pH, soil organic carbon content, soil total nitrogen content, the material of plastic, the addition rate of plastic, size of plastic, and experiment duration. Following these criteria, our database recorded 48 eligible independent articles (Text S1) and provided a total of 710 unique experimental comparisons (for PRISMA flow diagram please see Supplementary Figure S1).

### 2.2. Statistical analysis

The natural log-transformed response ratio (lnRR) was calculated to evaluate the responses of soil microbial parameters and other related soil properties related to the effects of plastic between control and treatment, which is unaffected by study design. The effect size of plastic was calculated as follows:


lnRR=lnxtxc=lnxt−lnxc

where x_c_ and x_t_ are the mean values of index x from the control (without plastic) and treatment (with plastic), respectively. The variance (v) of lnRR was then calculated as follows:


$$v=\frac{s_{t}^{2}}{n_{t}x_{t}^{2}}+\frac{s_{c}^{2}}{n_{c}x_{c}^{2}}$$

where n_c_ and n_t_ are the replication times of the control (without plastic) and treatment (with plastic), respectively; s_c_ and s_t_ are the standard deviations of the control (without plastic) and treatment (with plastic), respectively. If the studies did not report standard deviations, we calculated the average coefficient of variation (CV) within each observation and then approximated the missing standard deviations by multiplying the reported mean by the average CV (Van Groenigen et al., 2011; Li Y. et al., 2022).

With the development of high-throughput sequencing technology, the β-diversity of soil microbial community can be calculated and visualized by ordination plots (Anderson et al., 2011), which display microbial β-diversity within each treatment and the community structure differences among treatments. For the effect of plastic on soil microbial β-diversity and community structure, the first two ordination axes of ordination plots (including principal component analysis, principal coordinate analysis, redundancy analysis, canonical correlation analysis, and nonmetric multidimensional scaling, and so on) were extracted and transferred into one-dimensional data (Zhou et al., 2020). Briefly, the exact coordinates of samples were extracted to calculate the Euclidean distances among them using the ‘vegan’ package. Then, the RRs of microbial community β-diversity (lnRRb) and structure (lnRRs) were calculated as follows:


lnRRb=lnDtDc=lnDt−lnDc

lnRRs=lnDbDc+Dt=lnDt−lnDc+Dt

where D_c_, D_t_, D_b_, and D_c_ + D_t_ are the mean values of the distance within the control (without plastic), within the treatment (with plastic), between the control and treatment, and overall Dc and Dt, respectively. Microbial richness and Shannon index are widely used and highly recommended for representing microbial α-diversity (Fierer and Jackson, 2006). The present meta-analysis recorded four common microbial α-diversity metrics, namely the Shannon index, Chao1 index, ACE index, and OTU numbers. The Chao1 index, ACE index, and OTU numbers therein were then merged into a variable to represent microbial richness. The calculation and presentation of microbial richness, biomass, and functional variables were performed according to the well-established data syntheses (Chen et al., 2015; Luo et al., 2018; Kim et al., 2020; Zhou et al., 2020). The soil functionality was synthesized from 16 microbial indicators from 5 aspects, driving soil biogeochemical cycling and are frequently used to estimate the ecosystem functionality (De Souza Machado et al., 2018; Zhou et al., 2020; Lozano et al., 2021). The detailed methods are included in the Supplementary material.

The mixed-effect models were used to test whether plastic effects changed with the ecosystem and plastic types. If the 95% confidence interval (CI) for the effect size of the parameter does not overlap with zero, then the treatment (with plastic) is considered to have a significant effect (increased or decreased) relative to the control (without plastic). In addition, to compare the heterogeneity among subgroup categories, the between-group heterogeneity (Qm) was calculated and only the parameters with more than 5 observations in the category were included in these analyzes. In addition to the variables reported in the original articles, we also divide and define these variables again and incorporate them into the random forest models: for the categories in plastic amount (categorical), we classified the amount into low (< 10 g/kg, i.e., < 1%) and high (> 10 g/kg, i.e., > 1%) (Gao et al., 2021). In addition to the specific plastic type reported in the original articles, we further classified the plastic materials into aliphatic (PP, polypropylene; PE, polyethylene, including high-density polyethylene and low-density polyethylene) and others (plastics contain other functional groups, e.g., PET, polyethylene terephthalate; PS, polystyrene; PVC, polyvinyl chloride) as general plastic type (Wright et al., 2021). The incubation time (categorical) was classified as short (≤7 days) and long (>7 days; Wright et al., 2021). The ecosystems to which soils belong were classified into bare soil, cropland soil, grassland soil, and forest soil (Zhou et al., 2020).

To identify the key drivers that were most associated with soil microbial parameters in response to the plastic, feature selection was performed using two types of machine learning based on different constructing strategies: one with bagging decision tree (i.e., random forest analysis) while another one with boosting decision tree (i.e., aggregated boosted tree analysis); the two decision tree-based algorithms are powerful tools to detect the explanatory variables that are most related to the dependent variables, and are also nonparametric and nonlinear statistical methods with no prior distributional assumptions (Liu Y. X. et al., 2021). We introduced plastic type, amount, size as well as incubation time into the models in both continuous and categorical variable forms (Wright et al., 2021). The former strategy was performed in the package “rfPermute,” and the latter strategy was performed in the package “gbm.” For the random forest analysis, the importance of predictors was identified based on the “increase in mean square error” values, while for the aggregated boosted tree analysis, the importance of predictors was identified based on the “relative influence” values. Moreover, by incorporate the weight of sample to the bootstrap sampling, the weight was also taken into consideration when perform random forest analysis with bootstrapped preselection to identify the relative importance of all potential factors using the package “metaforest” (Abalos et al., 2022).

### 2.3. Model fitting and selection

We fitted both linear and nonlinear regressions to investigate the relationships between microbial parameters and plastic size, amount, and incubation time. For the linear regression, we used ordinary least squares regression (without considering the weight of the sample) and weighted least squares meta-regression (considering the weight of the sample) to assume that the response of microbial parameters to explanatory variables is gradual. If this is not the case, we then used general additive model with smoothing parameters to describe the nonlinear patterns (Hao et al., 2021). The AIC values of the models were then used to determine the best-fit-model. If the value of a general additive model was 2 units less than that of an ordinary least squares regression model, the former one would be selected as the final model (Hou et al., 2021); otherwise, the latter one was selected. The thresholds of a broken-stick model can be further explored only when the nonlinear model is suitable (Berdugo et al., 2020). Thus, we performed piecewise regression, an effective model to explore the threshold where two lines are joined (Toms and Lesperance, 2003). This analysis was conducted in the package “segmented,” and the model formula was expressed as:


$$Y=\beta_{0}+\beta_{1}\left(X\right)+\beta_{2}{\left(X-c\right)}^{*}+\varepsilon$$

where Y and X were the dependent variable and predictor, c and ε were the infection point and residual standard error. The part marked with asterisks was valid when the value of X > c , i.e., larger than the infection point.

### 2.4. Systematic review for network properties

To further explore the response of soil microbial community network patterns to plastics, we performed a systematic review of network properties due to the lack of quantifiable data. The detailed information for the systematic review of network properties is specified in Supplementary material.

### 2.5. Publication bias

The results suggested that most involved variables had nonsignificant publication bias in our meta-analysis (Supplementary Table S2; Supplementary Figure S2). The analysis and results of publication bias are detailed in Supplementary material.

## 3. Results

### 3.1. Overall response of soil microbial parameters to plastics

**Figure 1:**
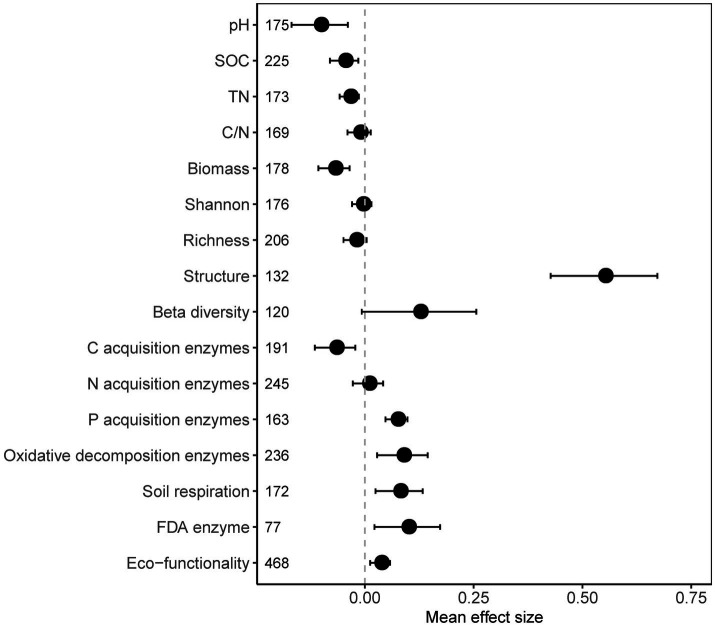
Overall response of the selected soil properties and microbial parameters to plastic residue. The mean bar values expressed as the mean effect size of each variable with 95% confidence intervals (CIs). The sample size of each parameter is given in parentheses.

In general, the presence of plastic in the soil has a considerable impact on the microbial community and soil properties. Soil pH, soil organic carbon (SOC), total nitrogen (TN), and microbial biomass was decreased significantly with plastics (Figure 1). Although microbial community diversity (both α- and β-diversity) did not change significantly, community structure was significantly changed. Synthesized from 16 individual soil microbial functional parameters, the soil microbial functionality significantly (*p* = 0.002) increased due to plastic (Figure 1), mainly driven by the enhancement of FDA enzymes (*p* = 0.011), soil respiration (*p* = 0.004), P-acq activity (*p* < 0.001), and OX activity (*p* = 0.003), whereas the C-acq activity significantly (*p* = 0.004) inhibited by plastic (Figure 1).

The systematic review of the microbial co-occurrence network’s response to plastics revealed that the topological properties of the network responded to it in diverse ways (Table 1). Uncertainty exists on the direction of the response for node and edge numbers, while the proportion of positive edges increased in most cases and the average path length decreased across all studies (Table 1).

**Table 1:** The effects of plastics on the topological properties of soil microbial co-occurrence networks.

Plastic type	Plastic amount (g/kg)	Plastic Size (μm)	Control settings	Descriptions in the original article	Node	Edge	Proportion Pos_edge	Average path length	Clustering coefficient	Average degree	Modularity	Reference
PLA	20	35	Without any amendment	The network was smaller, but more connected and complex, increased the density of connections and created more intricate network patterns	−	+	−					Chen et al. (2020)
PLA	20	35	Amended with rice straw	Decreased connections in the network, the links were consistently weaker	+	−	+					Chen et al. (2020)
PE	10	125	Amended with ciprofloxacin	Showed the nonrandom structure of microbial assembly		*n*	+	−	−			Wang et al. (2020)
PE	20	200	Without any amendment	Formed more tightened associations, decreased the density of connections and created less complex network patterns.	−	−	+	−	+	−	*n*	Rong et al. (2021)
PE	70	200	Without any amendment	Formed more tightened associations, decreased the density of connections and created less complex network patterns.	−	−	+	−	−	−	+	Rong et al. (2021)
PS	0.005	0.5	Without any amendment		*n*	+		−	+	+	−	Xu et al. (2021)
PS	0.005	0.5	Amended with sulfamethazine	Combination of sulfamethazine and PS gave a stronger “small world” topology than sulfamethazine	*n*	+		−	+	+	+	Xu et al. (2021)

The symbols indicated the plastics on properties; +: increased; −: decreased; n: no response.

### 3.2. The major predictor for responses of microbial parameters induced by plastics

**Figure 2:**
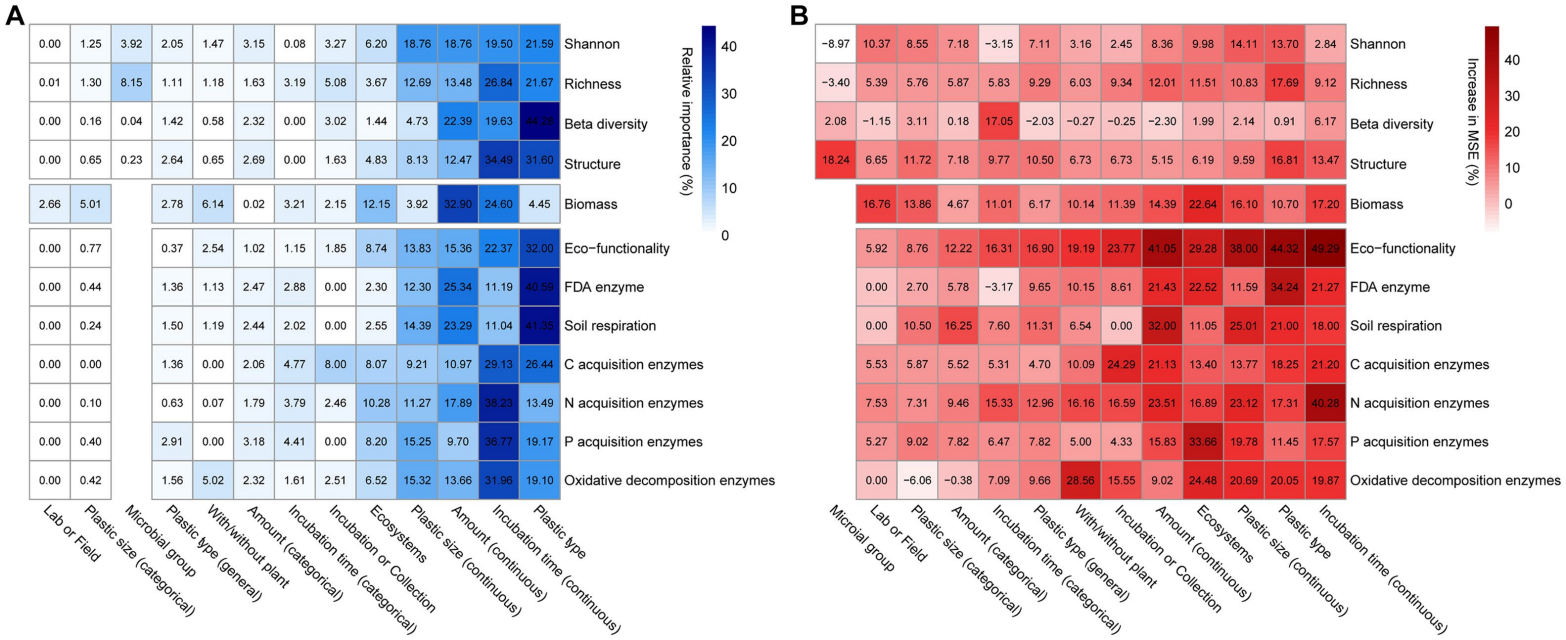
The influence of selected explanatory variables on each soil microbial parameter based on the machine learning approaches. The relative importance of explanatory variables to response variables revealed by the **(A)** aggregated boosted tree analyzes were showed in blue fine-grained heatmap. The increase in mean square error (MSE) of explanatory variables to response variables revealed by the **(B)** random forest analyzes were showed in red fine-grained heatmap. The observations for the machine learning models in predicting variables from Shannon to Oxidative decomposition enzymes are: 153, 182, 72, 72, 97, 402, 72, 143, 184, 206, 130, and 203.

Both the random forest and aggregated boosted tree machine learning algorithms showed that several factors related to ecosystem type, plastic properties and incubation time were the major predictor affecting the response of soil microbial community parameters (Figure 2). Specifically, ecosystem type, incubation time (continuous), plastic amount (continuous), plastic size (continuous), plastic type had the largest impact on microbial communities, while the effect sizes on these parameters showed no detectable variations across microbial groups (Figure 2). The results of random forests demonstrated that the effects of plastics on microbial parameters were mostly explained by plastic type, size, amount, and incubation time across a wide range of experimental and plastic factors (Supplementary Figure S5).

### 3.3. The effect of plastic amount on the response of soil microbial parameters

**Figure 3:**
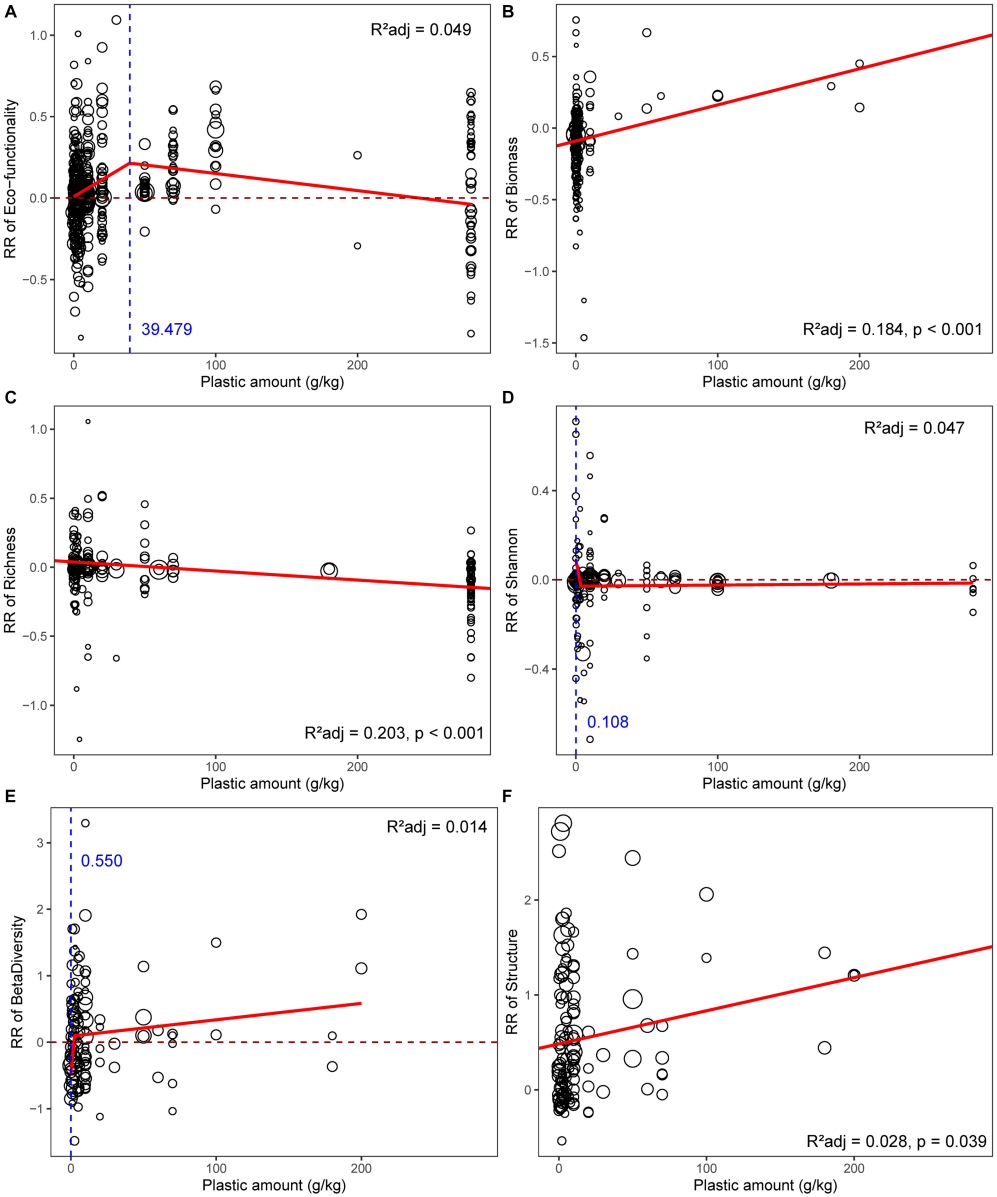
Relationships between plastic amount and response ratio of soil microbial **(A)** functionality, **(B)** biomass, **(C)** richness, **(D)** Shannon index, **(E)** β-diversity, **(F)** community structure. The red solid line represents the fitted significant (*p* < 0.05) weighted linear model or broken-stick model, while the blue dashed line represents the infection point of broken-stick model. The sizes of dots were proportional to the weights of samples.

The effect of plastics on soil microbial communities was modulated by plastic amount (Figure 3). The hormetic dose–response relationships were detected between plastic amount and soil functionality (Figure 3A), the response ratio of microbial functionality increased with the plastic amount from 0 to *ca.* 40 g/kg; the effect above this infection point was also positive but gradually declining, driven by the sharp rise in microbial activity indicators like FDA enzyme and soil respiration (Table 2). The response of biomass was significantly positively connected with plastic amount (Figure 3B), and the response of community structure also changed more drastically when more plastics were added (Figure 3F). Richness, on the other hand, showed a negative correlation with the plastic amount, indicating that more plastic addition had a detrimental influence on community α-diversity to a greater extent (Figure 3C). None of the unweighted linear model, weighted linear model, GAM, or broken-stick model can adequately describe the connection between plastic amount and Shannon index (Figure 3E; Supplementary Table S3). Specifically, the response of microbial diversity, biomass, and functionality was unaffected by the nanoplastic quantity (*p* > 0.05; Supplementary Figure S6).

**Table 2:** Linear regression analysis between characteristics of plastic residues with different soil microbial parameters under plastic residue.

Microbial parameters	Incubation time (continuous)					Amount (continuous)					Plastic size (continuous)				
	*R* ^2^	*p*-val	*n*	Intercept	Slope	*R* ^2^	*p*-val	*n*	Intercept	Slope	*R* ^2^	*p*-val	*n*	Intercept	Slope
Richness	−0.004	0.604	199			**0.089**	**<0.001**	**203**	**0.023**	**−**	−0.005	0.915	192		
Shannon	−0.006	0.938	170			−0.005	0.724	174			**0.019**	**0.043**	**162**	**−0.002**	**−**
Beta diversity	−0.002	0.383	94			0.126	0.126	118			−0.009	0.751	106		
Structure	−0.007	0.593	106			**0.031**	**0.025**	**130**	**0.455**	**+**	**0.028**	**0.038**	**118**	**0.578**	**−**
Biomass	0.009	0.157	112			**0.082**	**<0.001**	**175**	**−0.121**	**+**	0.008	0.167	118		
Eco-Functionality	0.005	0.084	409			0.003	0.117	464			−0.002	0.632	437		
FDA enzyme	**0.010**	**0.010**	**76**	**<0.001**	**+**	**0.327**	**<0.001**	**76**	**−0.013**	**+**	0.035	0.057	76		
Soil respiration	**0.030**	**0.021**	**144**	**0.208**	**−**	**0.482**	**<0.001**	**171**	**−0.041**	**+**	**0.075**	**<0.001**	**144**	**0.169**	**−**
C acquisition enzymes	**0.016**	**0.046**	**184**	**−0.033**	**−**	**0.171**	**<0.001**	**189**	**0.071**	**−**	**0.053**	**0.001**	**189**	**−0.075**	**+**
N acquisition enzymes	0.009	0.087	213			**0.044**	**0.001**	**241**	**0.055**	**−**	**0.022**	**0.012**	**241**	**−0.001**	**+**
P acquisition enzymes	**0.058**	**0.003**	**137**	**0.050**	**+**	**0.069**	**<0.001**	**161**	**0.047**	**+**	0.002	0.249	161		
Oxidative decomposition enzymes	**0.094**	**<0.001**	**208**	**−0.020**	**+**	**0.051**	**<0.001**	**232**	**0.165**	**−**	0.004	0.169	232		

The minus sign denotes a negative linear relationship, while plus sign represents a positive linear relationship.Linear regression analysis between characteristics of plastic residues with different soil microbial parameters under plastic residue. The values of significant relationships are bolded.

### 3.4. The effect of plastic size on the response of soil microbial parameters

**Figure 4:**
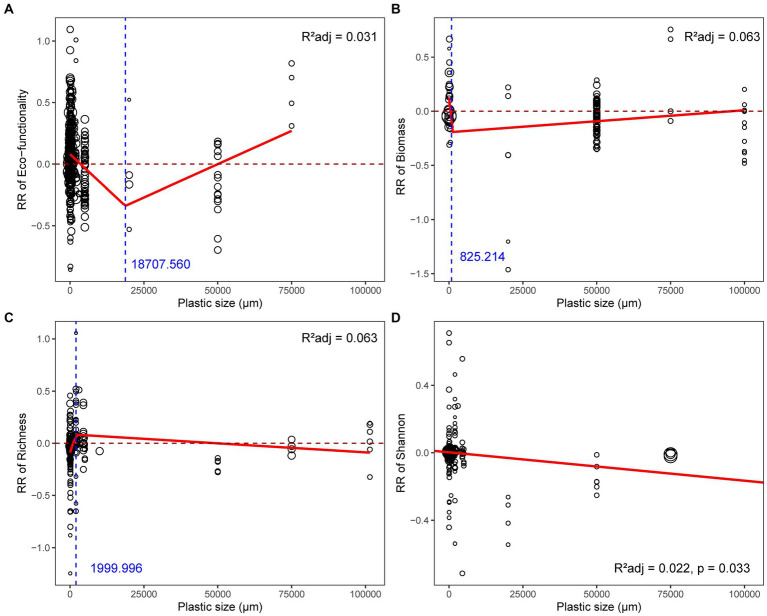
Relationships between plastic size and response ratio of soil microbial **(A)** functionality, **(B)** biomass, **(C)** richness, **(D)** Shannon index. The red solid line represents the fitted significant (*p* < 0.05) weighted linear model or broken-stick model, while the blue dashed line represents the infection point of broken-stick model. The sizes of dots were proportional to the weights of samples.

The threshold plastic size at which the response of microbial functionality turned from positive to negative was around 3564.78 μm, and the infection point where the slope changed was 18707.56 μm (Figure 4A). The consistent negative impacts of plastics on microbial Shannon index and biomass appeared in plastic sizes spanning numerous orders of magnitude, from nanometers to centimeters, even if the best-fit models varied (Supplementary Table S3; Figures 4B,D). Additionally, the response of richness increased throughout a small range of nanoplastic sizes (Supplementary Figure S7C; Figure 4C).

### 3.5. The effect of plastic type on the response of soil microbial parameters

Different types of plastics made from different materials also caused microbial community parameters to respond variously. The microbial α-diversity was significantly decreased by the PE and PVC (p_richness_ < 0.001 for PE; p_Shannon_ = 0.006 for PVC). The microbial biomass (*p* = 0.002) and C-acq activity (*p* < 0.001) were significantly reduced under PE; in contrast, PP induced a significant increase in microbial α-diversity (*p*_richness_ < 0.001) and in all functional parameters (Supplementary Figure S8A). Meanwhile, the degradable plastic, polycaprolactone (PCL), and polylactic acid (PLA) caused no significant changes in microbial diversity, structure, and specific functionalities (Supplementary Figure S8A).

### 3.6. The effect of incubation time and ecosystem type on the response of soil microbial parameters

The response of microbial functionality caused by plastics gradually decreased over time, reaching the lowest at the infection point at 75 days, suggesting that the positive effects of plastics on soil microbial functionality firstly disappeared over time, and then recovered and continued to increase (Supplementary Figure S9A). Once the plastics were incorporated, the early and middle stages of incubation showed a negative response in terms of microbial biomass and α-diversity, these effects reached the infection points at 95 days, *ca* 45 days, and 60 days, and there was a tendency to produce a positive response in the late stage (Supplementary Figures S9B–D).

The impact of plastics differs among ecosystem types. Microbial community parameters showed the most sensitive response for studies incorporating residues into cropland soils (Supplementary Figure S8B). The changes in microbial richness indicated a decline in community species (*p* < 0.001); the absolute value of the response ratio of richness was always greater than that of the Shannon index (Supplementary Figure S8B). Microbial biomass in cropland soils also decreased significantly (*p* < 0.001). In cropland soils, plastics significantly decreased microbial C-acq activity, while soil respiration and P-acq activity were significantly increased; these were not observed in other ecosystems. Correspondingly, this parameters in grassland soils responded stably to plastics (Supplementary Figure S8B).

### 3.7. Changes of soil properties altered the response of soil microbial parameters to plastics

Plastics could also affect soil microbial communities by changing soil physiochemical properties. The change of pH, response ratios of SOC and TN were closely related to several microbial parameters (Supplementary Figure S10). Additionally, the change of these three soil physiochemical parameters was significantly related to microbial functionality (Supplementary Figures S10A,D,G), their relationships with biomass were also similar (Supplementary Figures S8E, S9H, S10B). The response ratio of SOC and that of richness was synchronized (Supplementary Figure S10F). However, the correlations between the change of pH, response ratios of SOC and TN to other microbial parameters such as Shannon index, community structure were insignificant (*p* > 0.05; Supplementary Figures S11–S13).

### 3.8. Relationships among microbial diversity, community structure, biomass and function as affected by plastic

The relationships among microbial diversity, community structure, biomass and function as affected by plastic were revealed by linear regression (Supplementary Figure S14). The response ratio of microbial diversity had a significant positive correlation with biomass, mirroring microbial biomass production. A significant positive correlation existed between the response ratio of the Shannon index and community structure and indicating that changes in the relative abundance of taxa shaped the community structure. Importantly, no significant relationship was observed between the ratio of microbial functionality and community structure or diversity (Supplementary Figure S14).

## 4. Discussion

### 4.1. Overall effects of plastics on soil microbial parameters

Consistent with the meta-analysis results of multiple global change factors (Zhou et al., 2020), plastics had no negative impact on soil microbial α-diversity, such findings diverge partly from hypothesis H2. In conjunction with distinct changes in microbial community structure, this might be explained by the adaption of microbes to disturbances like plastics. As a result of plastic incorporation, the negative impacts caused by soil physical changes like moisture and porosity, as well as soil chemical changes like the release of chemical additives, lead to the extinction of microbes that cannot adapt to the present environment (You et al., 2022). Conversely, the formation of plastisphere, a new and unique microbial habitat, stimulates the enrichment of plastic-degrading microbes and pathogens, which may increase the microbial diversity in specific spaces (Liu M. et al., 2023). Thus, the compensation of these two opposite effects may cancel out each other, eliminating any significant effect of plastics on soil microbial α-diversity.

As a new and formerly neglected input of organic carbon from fossil sources (Rillig, 2018; Mbachu et al., 2021; Rillig et al., 2021), plastic does not stimulate microbial biomass but significantly decreases it (Figure 1). The increased microbial functionality was also reflected in the oxidation of substances, including (1) depolymerization of lignin-like substrates and hydrogen peroxide degradation; (2) carrying out the enzymatic reactions degrade plastic derivatives into oxidative metabolites after they were transported to microbial cells (Amobonye et al., 2021). These processes are energy-consuming processes that require high energy and carbon costs (Bonner et al., 2019). Notably, as enzyme activities only represent potential rates depending on the specific experimental conditions, they must be interpreted with caution (Hazard et al., 2021).

Recent meta-analyzes focused on microplastics indicate that microbial biomass significantly increased by it, and attributed this phenomenon simply to the fact that microplastics, being carbon-based materials, become soil carbon components and can be utilized by microbes (Li H. et al., 2022; Zhang J. et al., 2022; Liu M. et al., 2023). The microbial biomass response may no longer be positive, though, when the impacts of plastics are considered across a larger size range (Zhang J. et al., 2022; Zhang et al., 2022a). Our results, which incorporated more data and studies, supported this conclusion and showed that the response may potentially be negative. This is because: (1) larger-sized plastics behave differently in the environment to microplastics, particularly the plastic debris left behind by the use of film in farmlands. They have smaller specific surface areas and are less likely to come into touch with the “pioneer” microbial taxa that degrade plastics, and the negative effect on the soil microbial habitat is also greater. Additionally, years of continued usage enhanced the negative impacts, particularly the enrichment and accumulation of heavy metals, pesticides, and phthalate esters, all of which have negative effects on microbial biomass (Wang et al., 2016); (2) smaller-sized nanoplastics may directly damage microbial cells, resulting in reduced biomass (Awet et al., 2018). Back to the perspective of microbial degradation and utilization of plastics, in fact, unlike the carbon substrate input in the traditional understanding, plastics are extremely difficult to degrade and only a small fraction of derived carbon is microbial available (Xiao et al., 2022), the microbes cannot obtain a sufficient amount of substrate to support growth, resulting in a decrease in microbial biomass. Microbes capable of further degrading the recalcitrant carbon components of plastics have so far only been identified at the strain level (Gambarini et al., 2021), their increased absolute abundance cannot drive the patterns of whole community biomass. In addition, functional complexity, such as the molecular diversity of carbon compounds, increases SOC persistence, making it likely more difficult for microbes to process organic carbon (Lehmann et al., 2020). Overall, microbes seem to be “deceived” by plastics, with the exhaustion of bio-labile carbon substrates, the microbial carbon use efficiency declined (Zang et al., 2020), and increased oxidase activity related to toxicological metabolism and recalcitrant carbon decomposition implies a large consumption of energy (Figure 1).

The interactions between community members are represented by the microbial co-occurrence network, and mounting evidence suggests that the network’s properties can also characterize how the community reacts to disturbances in the soil, such as plastics (Shi J. et al., 2022). The microbial co-occurrence networks could indicate the potential interconnections between them, and the average path length and proportion of negative correlations are important indicators of network stability (Faust and Raes, 2012; De Vries et al., 2018). Both of these two indicators were decreased by plastics, imply that such disturbance weakened microbial network stability and makes it more sensitive to environmental changes. Small-world networks emerged as a result of the loss or replacement of keystone taxa, altering the relationships and patterns of microbial interaction. As a result, the amendment of plastics reduced network stability and increased the vulnerability of microbes to external disturbance.

### 4.2. Plastic property-dependence of the responses of soil microbial parameters

Our findings demonstrated that plastic type, amount, and size affect the magnitude and direction of microbial responses to plastics, supporting hypothesis H1. Notably, a linear relationship cannot be established for a considerable number of comparisons, and piecewise regression performed better at explaining the patterns. Our results are more in line with reality, indicating that plastics have complicated effects on microbial parameters and that there may be dose and size thresholds or infection points rather than sustained changes to these parameters (Agathokleous et al., 2021b).

The plastic materials differ substantially, and these differences lead to distinct responses of microbial communities. Similar to the results derived from intertidal marsh sediments (Seeley et al., 2020), the PE and PVC in soils also inhibited the soil microbial community the most. PP had a noticeable promotion effect on microbial α-diversity and all ecological functions. This may be related to the chemical properties of PP, which has a methyl group side-branched chain and is more prone to aging and degradation to produce low-molecular-weight degradation products; the degradation period is a fraction or even tenth of that of PVC and PE (Zhang S. L. et al., 2021). PLA and PCL, the two degradable plastics involved in the current study, did not cause significant changes in microbial parameters when incorporated into the soil. There are also potentially negative effects of degradable plastics that are easily overlooked: degradable plastics are more likely to interfere with the formation of macro-aggregates, with stronger negative effects on soil aggregates and nutrients than non-degradable plastics (Zhao et al., 2021). Additionally, the depolymerization and hydrolysis of PLA release lactic acid and decrease pH, which may negatively affect microbes (Karamanlioglu and Robson, 2013). Hydrophobic PCL is generally mixed with hydrophilic starch into plastics such as agricultural film, but as starch is easily utilized by microbes, the crystallinity of PCL increases and the enzymatic depolymerization decreases (Tokiwa et al., 2009).

Smaller plastics have more adverse toxicological effects on larger organisms (Wan et al., 2023), since microplastics may enter plant tissues through root pores or enter animal bodies through ingestion, tend to be more harmful to plants and soil animals than larger-sized plastics (Li et al., 2020; Wang et al., 2022b,d). Yet microbial responses to plastics are reflected at the level of the entire community (Shafea et al., 2023), hence, this rule may not always be accurate when extrapolating to the effect of entire plastics to microbial communities (Mammo et al., 2020; Wiedner and Polifka, 2020; Zhang S. L. et al., 2021). Taking into account all plastic residues, it appears that the effects of plastics on microbial parameters have a size threshold. The threshold for soil biological indicators such as soil respiration is 500 μm, and plastics bigger than this will cause the response ratio to decline (Liu X. et al., 2023). The current study showed that the threshold size for reducing microbial functionality due to plastics was greatly extended to *ca* 3,500 μm and also identified an inflection point where the direction of slope changed outside the range of microplastics. Changes in their environmental behaviors in soil were regulated by the effects of plastic size on microbial functionality. When the size was above its threshold, the colonization ability of microbes changed, which therefore directly affected the substrate utilization functions (Zhang et al., 2019). In particular, large- and medium-sized plastics may enrich carbon and nitrogen substrates through the surrounding soils rather than self-produced, forming new microbial activity hotspots that are conducive to biomass accumulation; the premise is the selection of microbes with high metabolic efficiency, so the continuous decrease of microbial diversity can be explained (McKay et al., 2022). Indirectly, the introduction of large- and medium-sized plastics altered soil aggregation and moisture status, which differed from the effects of microplastics and nanoplastics. As a result, microbial environmental adaption and activity were subsequently altered (Zhang et al., 2022a). A recent field study published after our literature survey revealed that plastics larger than this threshold size increase the activity of most extracellular enzymes (Fu et al., 2023). The inclusion of larger plastic sizes (especially mesoplastic and macroplastic) will considerably expand the information on the response of microbial communities. In particular, given that macroplastics entering soils are precursors of microplastics, understanding the impact of plastics above the size of microplastics on microbial parameters is critical for us to take timely measures based on their environmental behaviors. Interpret the size- dependent effects of nanoplastic on microbial richness should be with caution, since the sample size was limited in this study. Similarly, the observations belonging to meso- and macroplastics were also much less than microplastics, the infection points determined here only apply to the current data.

Previous studies that integrated individual microbial functions with plastic amounts suggested a linear-non-threshold model with varying directions (Qiu et al., 2022; Wan et al., 2023). Our more comprehensive investigation suggests the overall response of microbial functionality has thresholds and endpoints, which can better explain variations in soil functions in field studies (Xu B. et al., 2020). While the mechanisms are different, the responses of microbial functionality had a clear hormetic dose–response pattern, which was similar to how macroorganisms responded to plastics in terms of growth and reproduction (Agathokleous et al., 2021b). Larger organisms exhibit hormesis as a result of low-concentration leachates that cause physiological responses in living things, or plastics that trap concurrent pollutants and prevent biological contact (Agathokleous et al., 2021b). In contrast, the positive response of microbes attributed to more plastics allows soil microbes to come into extensive contact with them, the expanded plastic-soil-microbe interaction area facilitates microbes to form biofilms and stimulates their mobility, enzyme secretion, and respiration (Wan et al., 2023). The soil hydrothermal status under incubation experiment is constant, and the plastic amount threshold altered microbial functionality was 10 g/kg or 1 g/kg (Zhang et al., 2022b); accounting for environmental fluctuations under field conditions, our results suggest that the plastic amount threshold is even lower, and trace amounts are sufficient to cause significant effects (Shen et al., 2023). In addition, the increase of plastics causes continued accumulation of toxins such as phthalates (Xu B. et al., 2020), resulting in the selection of microbial community members, and the diversity responses continued to decline.

### 4.3. Potential mechanisms of changes in soil properties that alter microbial parameters

Supporting hypothesis H1, changes in soil physicochemical properties caused by plastics regulated microbial responses. The pH_pzc_ of plastics is 3.96–4.30, considerably lower than the soil pH (Torres et al., 2021), this deprotonates the hydroxyl groups on the plastic surface and lowers the environmental pH, which in turn reduces the microbial biomass due to niche limitation. The response of soil pH value is an important predictive factor for microbial parameters detected in the synthesis, consistent with other studies at local sites or large spatial scales (Rousk et al., 2010; Shi et al., 2018). Soil pH directly imposes physiological constraints on microbes by affecting membrane-bound proton pumps and protein stability, and indirectly affects microbes by spillover effects that control the availability of other elements (Lammel et al., 2018). As a result, when soil pH exceeds a particular range, it limits the net growth of individual taxa that cannot survive, exerts selection pressure on taxa that are less tolerant than alkaliphiles or acidophiles, and alters the outcome of competition (Booth, 1985), which causes changes in the microbial parameters. SOC and TN are essential indicators of soil fertility; a decline in soil carbon and nitrogen is typically one of the mechanisms generating a decrease in microbial biomass (Wang Y. et al., 2021), we also found that plastics have such impacts on soil biomass. Changes in soil physical factors, such as porosity, aggregation, aeration, and/or sorption and migration of chemicals and additives, are proposed to be linked to the effects of plastics on soil C/N dynamics (Lehmann et al., 2021; Riveros et al., 2022). Such variations may restrict the ability of microbes to acquire resources. Additionally, the negative impacts of plastics on plant litter may result in lower inputs of soil organic matter and further resource limitations, which in turn affect soil microbial communities, their activity, and with them the mineralization rates of soil organic matter (Zhou et al., 2021).

### 4.4. Decoupling of changes in microbial diversity and functionality with plastic

The relationships between microbial functions and corresponding microbes vary depending on the specific process. Microbial functional redundancy, i.e., the insurance effect, enables species with different niches or sensitivities to environmental stressors to perform the same function (Saleem et al., 2019). The scenario could be that plastics lead to the selection of soil microbial members and the replacement of niches results in unchanged species diversity. Some specific functions such as C-acq activity are reduced, as the functional redundancy of carbon transformations reduces with increasing carbon source recalcitrance, while the microbes that secreted these enzymes went extinct. However, functions such as respiration are the sum of multiple physiological processes, carried out by a large number of microbes, and the decrease in diversity will not lead to its reduction. Overall, supporting hypothesis H2, the effect of plastics on soil microbial diversity was decoupled from its effect on functionality. This is consistent with the findings of a large-scale study focus on the impacts of global change factors on microbial ecology, which showed widespread negative or decoupled relationships between these parameters (Zhou et al., 2020).

Plastics increased horizontal and vertical gene transfer among microbes (Arias-Andres et al., 2018; Rillig et al., 2019), then these gene exchanges jointly lead to a significant decoupling of microbial functions from their phylogenetic locations (Martiny et al., 2015; Louca et al., 2016). Thus, the changes in microbial community structure were also decoupled from microbial functionality as affected by plastics. Previous work indicated that microbial functional gene abundance is closely related to its functionality and called for further research on the relationships between particular enzyme activity and microbial functional genes (Chen and Sinsabaugh, 2021). Incorporating similar frameworks into research on the impact of plastic on soil microbial communities will further enhance the connection and comparability of this global change factor with others.

### 4.5. Limitations

The initial soil properties may determine the subsequent response of microbial properties to plastics. However, the lack of data prevented us from incorporating these indicators into machine learning models, and the high proportion of cultivation experiments made extracting data from global soil databases based on latitude and longitude unreliable. Additionally, the studies using high-throughput sequencing to explore the response of soil microbial communities to plastic are still scarce; articles included in the current meta-analysis rarely provide raw sequencing data or access numbers in publicly available repositories. This limits the feasibility of integrating samples from various studies for *de novo* raw data analysis. Moreover, the presence of a putative species in a sequencing assay does not necessarily equate to the organism being active. Last but not least, this study is a comprehensive meta-analysis derived from the published data, it is necessary to conduct subsequent laboratory or field studies based on the present results. In the future, the integration of plastic effects on the microbial community assembly processes, metabolic patterns, absolute quantitation of microorganisms, and other topics at the molecular ecology level deserve more attention.

## 5. Conclusion

**Figure 5:**
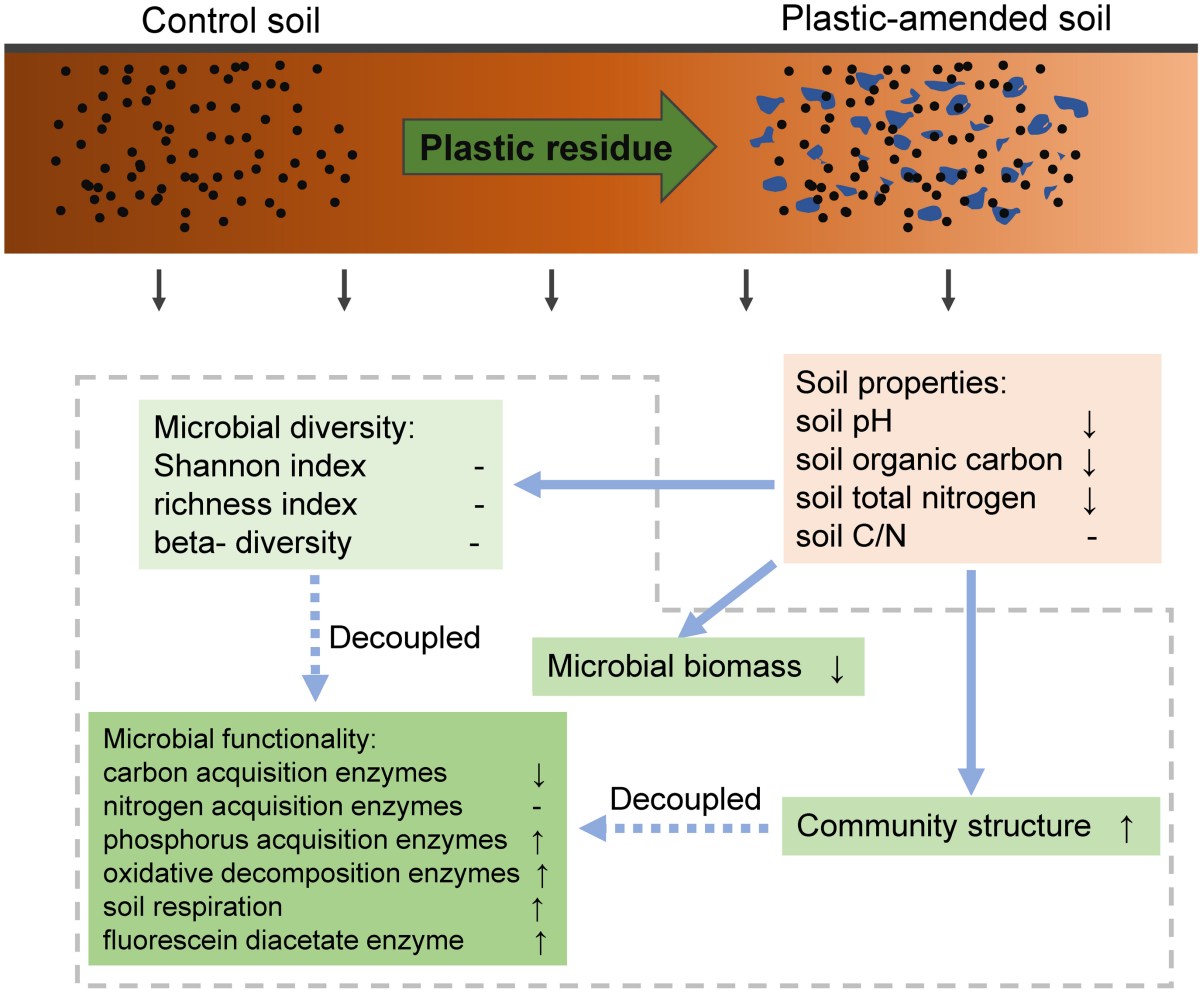
A conceptual diagram displayed the effects of plastic residue on soil microbiome, detailly including microbial diversity, biomass, and functionality. The up and down arrows indicate the significantly increasing and decreasing trends of the variables under plastic residual soils compared to controlled soils, respectively; while the crossbars indicate insignificant response of the variables.

In summary, this study indicates that plastics have significant impacts on soil microbial communities. Generally, it decreased microbial biomass but increased carbon mineralization. The degradable plastics cause no significant response in microbial parameters; however, PP and PE have positive and negative effects on microbial richness, respectively. Different from the linear response of larger organisms, the increase in plastic size has more adverse impacts on soil functionality, but the trend was the opposite until the infection point beyond the range of microplastics. The effect of the amount of plastic on microbial functionality fit the “hormetic dose–response” model, which was consistent with how larger organisms reacted to plastics. The changes in soil pH, SOC, and TN were in tandem with soil microbial functionality, biomass, and richness as affected by plastics. We also emphasize that as a global change factor that has recently been taken seriously, the changes in microbial diversity and structure were decupled from its functionality (Figure 5). In the context of persistent soil plastic pollution on a global scale, our results contribute to a comprehensive understanding of the underlying mechanisms of how it affects soil microbes, and future management measures should be taken to increase the organic carbon content to offset carbon loss due to microbial activities.

## Data availability statement

The datasets presented in this study can be found in online repositories. The names of the repository/repositories and accession number(s) can be found at: https://doi.org/10.6084/m9.figshare.22683682.

## Author contributions

YüL: Investigation, Methodology, Software, Writing – original draft. YH: Data curation, Methodology, Writing – review & editing. QH: Data curation, Formal analysis, Software, Validation, Writing – original draft. ML: Data curation, Validation, Visualization, Writing – review & editing. ZW: Funding acquisition, Project administration, Resources, Supervision, Visualization, Writing – review & editing. MR: Investigation, Methodology, Validation, Writing – review & editing. YuL: Funding acquisition, Methodology, Project administration, Resources, Software, Supervision, Validation, Writing – review & editing. TY: Funding acquisition, Project administration.
