# Supplementary material for: Soil microbial community parameters affected by microplastics and other plastic residues
Source: Front Microbiol. 2023 Oct 12;14:1258606. doi: 10.3389/fmicb.2023.1258606 (PMC10601715; doi:10.3389/fmicb.2023.1258606)
Supplement: Supplementary file 1 [file Data_Sheet_1.PDF]

Supplementary Information

For

"Soil microbial community parameters affected by microplastics and other plastic residues: A data synthesis

Yüze Li [a], Yuting Hou [a], Quanming Hou [a], Mei Long [a], Ziting Wang [a,b,c]*, Matthias C. Rillig [d,e], Yuncheng Liao [a]*

a College of Agronomy, Northwest A&F University, Yangling, 712100 Shaanxi, PR China

b College of Agronomy, Guangxi University, Nanning, 530004 Guangxi, PR China

c Guangxi Key Laboratory of Sugarcane Biology, Nanning, 530004 Guangxi, PR China

d Freie Universität Berlin, Institute of Biology, 14195 Berlin, Germany

e Berlin-Brandenburg Institute of Advanced Biodiversity Research (BBIB), 14195 Berlin, Germany

: Corresponding author: Ziting Wang; Yuncheng Liao
E-mail addresses:
zitingwang@gxu.edu.cn (Z.T. Wang); yunchengliao@163.com (Y.C. Liao).

This file includes:

- Supplementary Methods.
- Supplementary Tables S1-S5.
- Supplementary Figures S1-S12.
- Supplementary Text S1.

Supplementary Methods.

Synthesis of microbial parameters

We used the following proxies in order of availability to represent microbial biomass: chloroform fumigation indicators (microbial biomass carbon, MBC; microbial biomass nitrogen, MBN), phospholipid fatty acid (PLFA), and colony forming units (CFU). To avoid bias, the indicators of specific microbes like fungal hyphal length, bacteria 16s rRNA copy number, or arbuscular mycorrhizal fungal spores were excluded. The advantage of this way is that as many variables can be obtained to characterize the microbial biomass (Chen et al. 2018), facilitate the revealing of easily overlooked microbial biomass responses to plastics and its relationship to other indicators. We recoded 16 microbial functional variables related to soil biogeochemical cycling and microbial activity. In detail, these variables were: 1) soil microbial activity [soil respiration, fluorescein diacetate hydrolase (FDA) activity]; 2) soil carbon acquisition and cycling (C-acq) [α-1,4-glucosidase (AG) activity, β-1,4-glucosidase (BG) activity, β-D-cellobiosidase (CB) activity, β-1,4-xylosidase (XYL) activity, invertase (INV) activity]; 3) soil nitrogen acquisition and cycling (N-acq) [leucine aminopeptidase (LAP) activity, β-1,4-N-Acetyl-glucosaminidase (NAG) activity, urease (URE) activity]; 4) soil phosphorus acquisition and cycling (P-acq) [acid phosphatase (ACP) activity, alkaline phosphatase (ALP) activity, and recorded as phosphatase (PHOS) activity if not specify]; 5) soil oxidative decomposition (OX) [phenol oxidase (PO) activity, catalase (CAT) activity, and dehydrogenase (DHH) activity] (Table S1). These indices are good proxies of processes driving soil biogeochemical cycling, and are frequently used to estimate the contributions to ecosystem functionality of microbial communities under the effect of plastic (de Souza Machado et al. 2018; Lozano et al. 2021; Wang et al. 2021). Consider the response values of all relevant indicators (e.g. AG, BG, CB, XYL activity) as a dataset for a certain functional group (e.g. C-acq) and then calculate the overall mean effect size. Detailly, these variables were merged into parameters that represent the microbial C-acq enzyme activity, N-acq enzyme activities, P-acq enzyme activities, OX enzyme activities, and microbial community ecosystem functionality (Eco-fun), we used random-effect models to calculate the overall RR of multiple observed variables to calculate the above parameters for each observation, i.e., the weighted average value of different variables. Note that this method is slightly different from the averaging method to calculate ecosystem multifunctionality, the weight of the samples needs to be considered as the present data come from different articles. Similarly, the microbial richness was also generated from Chao1 index, ACE index, and OTU numbers in the same way as microbial functional variables.

Systematic review for network properties

The microbial co-occurrence networks are the characterization of microbial community stability and complexity, a comprehensive analysis of these could provide further insights into microbial responses to plastics. However, quantitative analysis of these indicators faces two difficulties. Firstly, the original sequencing data is highly inaccessible, and only three of the included articles deposited the data in public databases, which hinders data re-analysis. Secondly, only a few studies from four articles conducted microbial co-occurrence network analysis, and less data can be directly obtained. Hence, here we conducted a systematic review to qualitatively analyze the microbial co-occurrence network properties as affected by plastics, the trends of representative topological properties including node number, edge number, proportion of positive edges, average path length, clustering coefficient, average degree, and modularity were taken into consideration.

Publication bias

We used funnel plots and Rosenthal fail-safe numbers to assess the publication bias (Rosenberg 2005). The comparison between data from all cases and the aggregated weighted mean within-study suggests they were similar (Fig. S2), and the potential bias of non-independence is minimized (Lu et al. 2021; Nessel et al. 2021; Song et al. 2020). Then the sensitivity of the few variables with potential publication bias was then examined using the Trim–and–fill model (Duval and Tweedie 2000). After the adjustment, there were no significant differences in either direction or significance of results between simulated and observed effect size, i.e., no missing studies need to be added to the current database. The results derived from our multiple case studies analyses were similar to the aggregated results in these robustness tests. A fixed-effect model was used to test the difference in response ratio of the three microbial richness indexes, i.e., Chao1 index, ACE index, and OTU numbers, within each study (Zhou et al. 2020). A total of 100 observations reported at least two types of microbial richness metrics, of which only 6 showed significant differences (Fig. S3), indicating that the use of different microbial richness metrics would not introduce much bias in richness analysis. If only one index was reported, then it was treated as a proxy for richness. Otherwise, for observations with two or more richness indexes, we used a random-effect model to calculate the overall response of richness to plastics from these three indices. According to the results of these analyses, we did not find significant publication bias that might bias our results (Table S2). We also conducted “leave-one-out” sensitivity analysis to check the robustness of variables’ effect sizes of each study by function “leave1out” in package “metafor”. The results indicate that our effect sizes were robust (Fig. S4). The results of publication bias and sensitivity analysis provided convincing evidence that the current meta-analysis minimized the problems of nonindependence (Lu et al. 2021).

Table S1. Description of all included extracellular enzymes in the present meta-analysis. EC: enzyme commission number.

EC	Class	Extracellular enzyme	Abbreviation	Function
3.2.1.20	carbon acquisition	α-1,4-gulcosidase	AG	Hydrolysis of soluble saccharides
3.2.1.21	carbon acquisition	β-1,4-Glucosidase	BG	Hydrolysis of cellulose
3.2.1.91	carbon acquisition	β-D-cellubiosidase	CB	Hydrolysis of cellulose
3.2.1.37	carbon acquisition	β-1,4-xylosidase	XYL	Hydrolysis of xylan
3.2.1.80	carbon acquisition	Invertase	INV	Hydrolysis of sucrose
3.4.11.1	nitrogen acquisition	Leucine amino peptidase	LAP	Cleaving of peptide bonds in proteins
3.2.1.30	nitrogen acquisition	β-1,4-N-Acetyl-glucosaminidase	NAG	Hydrolysis of chitooligosaccharides
3.5.1.5	nitrogen acquisition	Urease	URE	Hydrolysis of urea
3.1.3.2	phosphorus acquisition	Acid phosphatase	ACP	Cleaving of PO43- from P-containing OM
3.1.3.1	phosphorus acquisition	Alkaline phosphatase	ALP	Cleaving of PO43- from P-containing OM 3
1.10.3.2	oxidative decomposition	Phenol oxidase	PhOx	Oxidation of lignin
1.11.1.6	oxidative decomposition	Catalase	CAT	Hydrolysis of hydrogen peroxide
1.1.1.1	oxidative decomposition	Dehydrogenase	DHH	Catalytic oxidation reduction reaction

Table S2. Results for the test of publication bias. k is the sample size for a variable. If the Rosenthal's fail-safe number (N) is larger than 5k+10, the result is robust regardless of any potential publication bias. The variables with potential publication bias were then examined using the trim-and-fill model. The model results showed that no significant differences in neither direction nor significance of results between simulated (after trim-and-fill) and observed (before trim-and-fill) effect size, i.e., no missing studies need to be added to the current database.

Variables	Number of observations (k)	Fail safe number (N)	Trim-and-fill analysis	
			Before Trim-and-fill	After Trim-and-fill
Microbial abundance				
allCFU	59	29358		
allPLFA	27	3536		
MBC	122	35796		
MBN	111	12381		
Microbial diversity and structure				
Shannon	176	44399		
OTUrichness	161	1099		
Chao1	125	64		
ACE	63	557		
Microbial eco-functionality				
Soilrespiration	172	36653		
FDA	77	8721		
AG	7	2436		
BG	143	611	0.039 (-0.144~+0.008)	0.011 (-0.007~+0.031)
CB	44	7530		
XYL	33	1549		
INV	66	184	-0.00045 (-0.019~+0.018)	0.012 (-0.007~+0.031)
LAP	20	40	0.117 (-0.011~+0.246)	-0.072 (-0.232~+0.088)
NAG	6	267		
URE	219	4182		
PHOS	44	9048		
ALP	21	270		
ACP	98	41933		
DHH	48	34839		
CAT	160	4287085		
PO	66	3217		
Soil properties				
pH	175	51563		
SOC	225	583329		
TN	173	45864		
C/N	170	28065		

Table S3. The best model for soil microbial parameter response for each predictor. Akaike information criteria (AIC) value was used to compare the fits of linear (OLS, ordinary least squares regression model), nonlinear (GAM, general additive model), and optimal threshold models (broken-stick model). Model with the lower AIC value has the best fits.

Microbial parameter	Predictor	Model	AIC	p-val	R2	Equation
Eco-functionality	Incubation time (continuous)	OLS	136.06	0.084	0.49%	
		GAM	**133.744**	0.051	1.93%	
		Meta-regression	/	0.185	0.64%	
		Broken-stick	/	/	0.73%	**Y= 0.058187-0.00055256X+0.00162460(X-75)**[*]
	Amount (continuous)	OLS	141.475	0.12	0.32%	
		GAM	**112.604**	<0.001	6.51%	
		Meta-regression	/	0.052	0.65%	
		Broken-stick	/	/	4.86%	**Y=0.0084596+ 0.0052173X-0.0010515(X-39.479)**[*]
	Plastic size (continuous)	OLS	151.193	0.632	-0.18%	
		GAM	**126.116**	<0.001	5.60%	
		Meta-regression	/	0.091	1.18%	
		Broken-stick	/	/	3.08%	**Y=0.079655-2.2345e-05X+1.0808e-05(X-18707.56)**[*]
Richness	Incubation time (continuous)	OLS	8.22149	0.604	-0.37%	
		GAM	**1.18978**	0.04	5.46%	
		Meta-regression	/	0.96	0.00%	
		Broken-stick	/	/	3.92%	**Y=0.042811-0.0036808X+0.0012913(X-45.468)**[*]
	Amount (continuous)	OLS	**-15.343**	<0.001	9.37%	
		GAM	/	/	/	
		Meta-regression	/	<0.001	20.31%	**Y=0.0382918-0.0006554544X**
		Broken-stick	/	/	/	
	Plastic size (continuous)	OLS	15.2909	0.915	-0.52%	
		GAM	**8.84277**	0.042	4.13%	
		Meta-regression	/	0.657	0.00%	
		Broken-stick	/	/	6.26%	**Y=-0.088096+8.5298e-05X-1.7313e-06(X-1999.996)**[*]
Betadiversity	Incubation time (continuous)	OLS	216.755	0.383	-0.25%	
		GAM	216.618	0.506	0.20%	
		Meta-regression	/	0.582	0.00%	
		Broken-stick	/	/	3.07%	Y=-0.68133+ 0.0254X-0.00030858(X-35)[*]
	Amount (continuous)	OLS	271.333	0.126	1.15%	
		GAM	271.229	0.19	1.45%	
		Meta-regression	/	0.067	3.43%	
		Broken-stick	/	/	1.38%	Y=-0.514940+7.0829X+0.002475(X-0.086)[*]
	Plastic size (continuous)	OLS	250.094	0.751	-0.86%	
		GAM	250.094	0.751	-0.86%	
		Meta-regression	/	0.735	0.00%	
		Broken-stick	/	/	0.15%	Y=-7.2688+1.3579e+01X-1.3388e-06(X-0.55)[*]

Microbial parameter	Predictor	Model	AIC	p-val	R2	Equation
Abundance	Incubation time (continuous)	OLS	28.5821	0.157	0.91%	
		GAM	**12.1164**	<0.001	16.50%	
		Meta-regression	/	0.498	0.00%	
		Broken-stick	/	/	7.35%	**Y=0.0070971-0.0016250X+0.0028067(X-95)**[*]
	Amount (continuous)	OLS	**47.2794**	<0.001	8.17%	
		GAM	/	/	/	
		Meta-regression	/	<0.001	18.36%	**Y=-0.08983879+0.002523488X**
		Broken-stick	/	/	/	
	Plastic size (continuous)	OLS	34.1407	0.167	0.78%	
		GAM	**0.54328**	<0.001	26.40%	
		Meta-regression	/	0.14	1.56%	
		Broken-stick	/	/	6.27%	**Y=0.12399-3.8323e-04X+2.0341e-06(X-825.214)**[*]
Shannon	Incubation time (continuous)	OLS	-113.26	0.937	0.59%	
		GAM	**-123.34**	0.005	5.67%	
		Meta-regression	/	0.549	0.00%	
		Broken-stick	/	/	8.22%	**Y=0.09649-0.0039792X+0.0020322(X-60)**[*]
	Amount (continuous)	OLS	-120.2	0.724	-0.51%	
		GAM	-117.66	0.331	2.80%	
		Meta-regression	/	0.636	0.00%	
		Broken-stick	/	/	4.70%	Y= 0.097581-1.1649e+00X+4.8939e-05(X- 0.108)[*]
	Plastic size (continuous)	OLS	**-107.68**	0.043	1.93%	
		GAM	/	/	/	
		Meta-regression	/	0.033	2.24%	**Y=0.002767009-1.683102e-06X**
		Broken-stick	/	/	/	
Structure	Incubation time (continuous)	OLS	225.22	0.593	-0.68%	
		GAM	223.287	0.25	1.78%	
		Meta-regression	/	0.562	0.00%	
		Broken-stick	/	/	2.03%	Y=0.32536+0.0026411X-0.0056749(X-120.885)[*]
	Amount (continuous)	OLS	**274.742**	0.024	3.12%	
		GAM	/	/	/	
		Meta-regression	/	0.039	2.28%	**Y=0.4844426+0.003493719X**
		Broken-stick	/	/	/	
	Plastic size (continuous)	OLS	**256.668**	0.0338	2.81%	
		GAM	/	/	/	
		Meta-regression	/	0.029	3.81%	**Y=0.6114128-6.445716e-06X**
		Broken-stick	/	/	/	

Table S4. Linear regression analysis between initial soil properties with different soil microbial parameters under plastic residue. pHi: initial soil pH; SOCi: initial soil organic carbon content; TNi: initial soil total nitrogen content. The minus sign denotes a negative linear relationship, while plus sign represents a positive linear relationship.

Microbial parameters	pHi					SOCi					TNi				
	R2	p	n	intercept	slope	R2	p	n	intercept	slope	R2	p	n	intercept	slope
Richness	-0.0050	0.6710	163			**0.1417**	**0.0000**	**139**	**0.05732**	**-**	**0.1584**	**0.0000**	**121**	**0.05185**	**-**
Shannon	-0.0071	0.8247	134			0.0054	0.2090	110			**0.0313**	**0.0475**	**94**	**0.01039**	**-**
Beta diversity	-0.0041	0.4668	113			-0.0088	0.6306	88			0.0103	0.2080	60		
Structure	-0.0030	0.4293	125			0.0021	0.2740	100			-0.0143	0.6946	60		
Biomass	**0.0464**	**0.0239**	**88**	**0.43918**	**-**	**0.1325**	**0.0010**	**71**	**-0.1176**	**+**	**0.4496**	**0.0000**	**59**	**-0.1626**	**+**
Eco-Functionality	**0.0134**	**0.0091**	**430**	**0.29023**	**-**	-0.0020	0.5797	351			-0.0018	0.4688	263		
FDA enzyme	**0.2247**	**0.0000**	**76**	**-0.9629**	**+**	**0.3289**	**0.0000**	**48**	**0.51208**	**-**	**0.5066**	**0.0000**	**67**	**0.77695**	**-**
Soil respiration	**0.1545**	**0.0000**	**144**	**-1.1422**	**+**	0.0056	0.2271	86			-0.0042	0.4588	106		
C acquisition enzymes	-0.0042	0.6300	182			-0.0053	0.9526	189			-0.0097	0.8225	99		
N acquisition enzymes	**0.0126**	**0.0468**	**234**	**0.22271**	**-**	**0.0216**	**0.0177**	**241**	**-0.035**	**+**	0.0178	0.0718	127		
P acquisition enzymes	-0.0030	0.4700	158			0.0002	0.3104	161			-0.0082	0.5413	77		
Oxidative decomposition enzymes	**0.1284**	**0.0000**	**232**	**1.39804**	**-**	**0.2049**	**0.0000**	**230**	**0.65407**	**-**	**0.3155**	**0.0000**	**98**	**0.44781**	**-**

Table S5. Linear regression analysis between change of soil properties with different soil microbial parameters under plastic residue. The minus sign denotes a negative linear relationship, while plus sign represents a positive linear relationship.

Microbial parameters	Change in pH					lnRR SOC					lnRR TN				
	R2	p	n	intercept	slope	R2	p	n	intercept	slope	R2	p	n	intercept	slope
Richness	-0.0045	0.4140	72			**0.1258**	**0.0003**	**91**	**-0.0715**	**+**	0.0088	0.2010	75		
Shannon	-0.0138	0.5380	45			0.0557	0.0829	37			-0.0500	0.9864	21		
Beta diversity	**0.0860**	**0.0208**	**50**	**-0.1209**	**-**	-0.0165	0.7260	54			**0.4788**	**0.0026**	**14**	**-0.4364**	**-**
Structure	**0.0532**	**0.0566**	**50**	**0.53189**	**-**	-0.0099	0.4960	54			0.0718	0.1726	14		
Biomass	**0.7309**	**0.0000**	**15**	**-0.1216**	**+**	**0.0792**	**0.0046**	**87**	**-0.0975**	**+**	**0.0387**	**0.0367**	**87**	**-0.0371**	**+**
Eco-Functionality	**0.2405**	**0.0000**	**102**	**0.04741**	**+**	**0.4463**	**0.0000**	**82**	**-0.0263**	**+**	**0.1911**	**0.0025**	**40**	**-0.1401**	**+**
FDA enzyme	/	/	/			/	/	/			/	/	/		
Soil respiration	0.7530	0.7530	5			/	/	/			/	/	/		
C acquisition enzymes	**0.1760**	**0.0046**	**38**	**-0.4187**	**+**	**0.4147**	**0.0000**	**58**	**-0.2618**	**+**	**0.2544**	**0.0005**	**40**	**-0.2744**	**+**
N acquisition enzymes	0.0234	0.1200	62			**0.1404**	**0.0012**	**64**	**-0.0151**	**+**	**0.1143**	**0.0174**	**40**	**-0.1499**	**+**
P acquisition enzymes	-0.0355	0.7450	26			0.1028	0.0652	24			/	/	/		
Oxidative decomposition enzymes	**0.1781**	**0.0000**	**96**	**0.06139**	**+**	**0.1069**	**0.0019**	**78**	**0.12462**	**+**	**0.3167**	**0.0002**	**36**	**-0.1872**	**+**

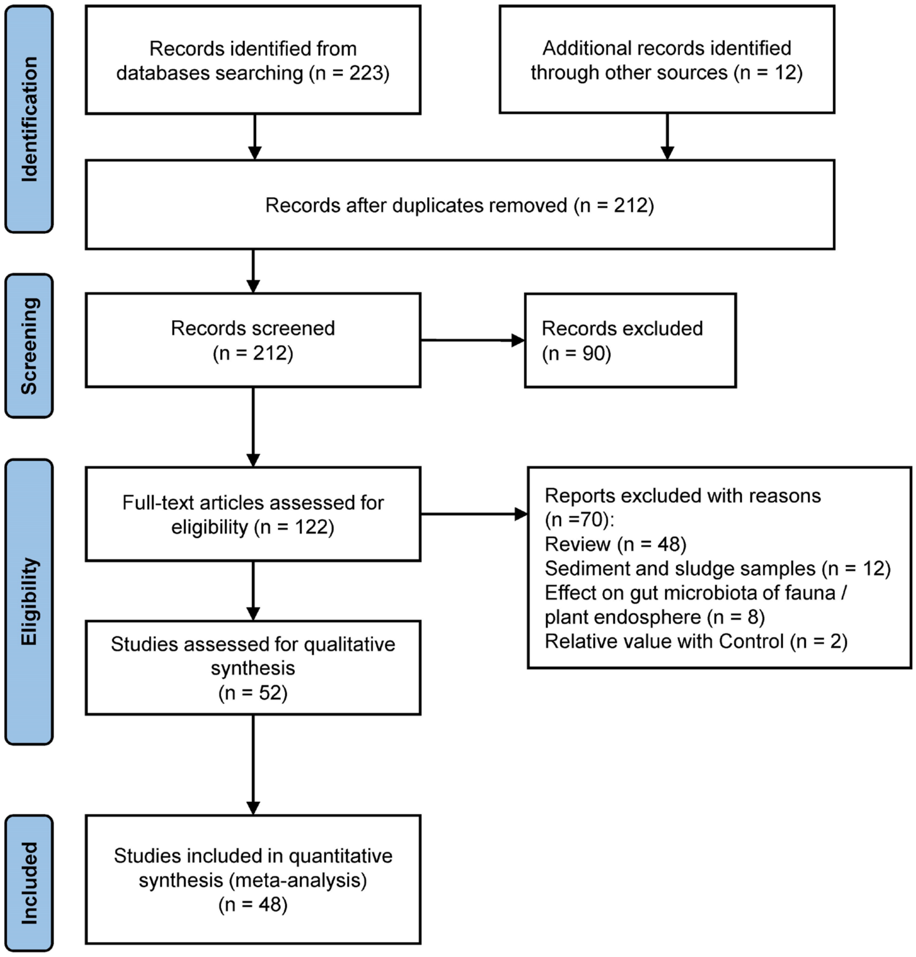

Fig. S1 PRISMA flow diagram showing the procedure used for selection of studies for synthesis.

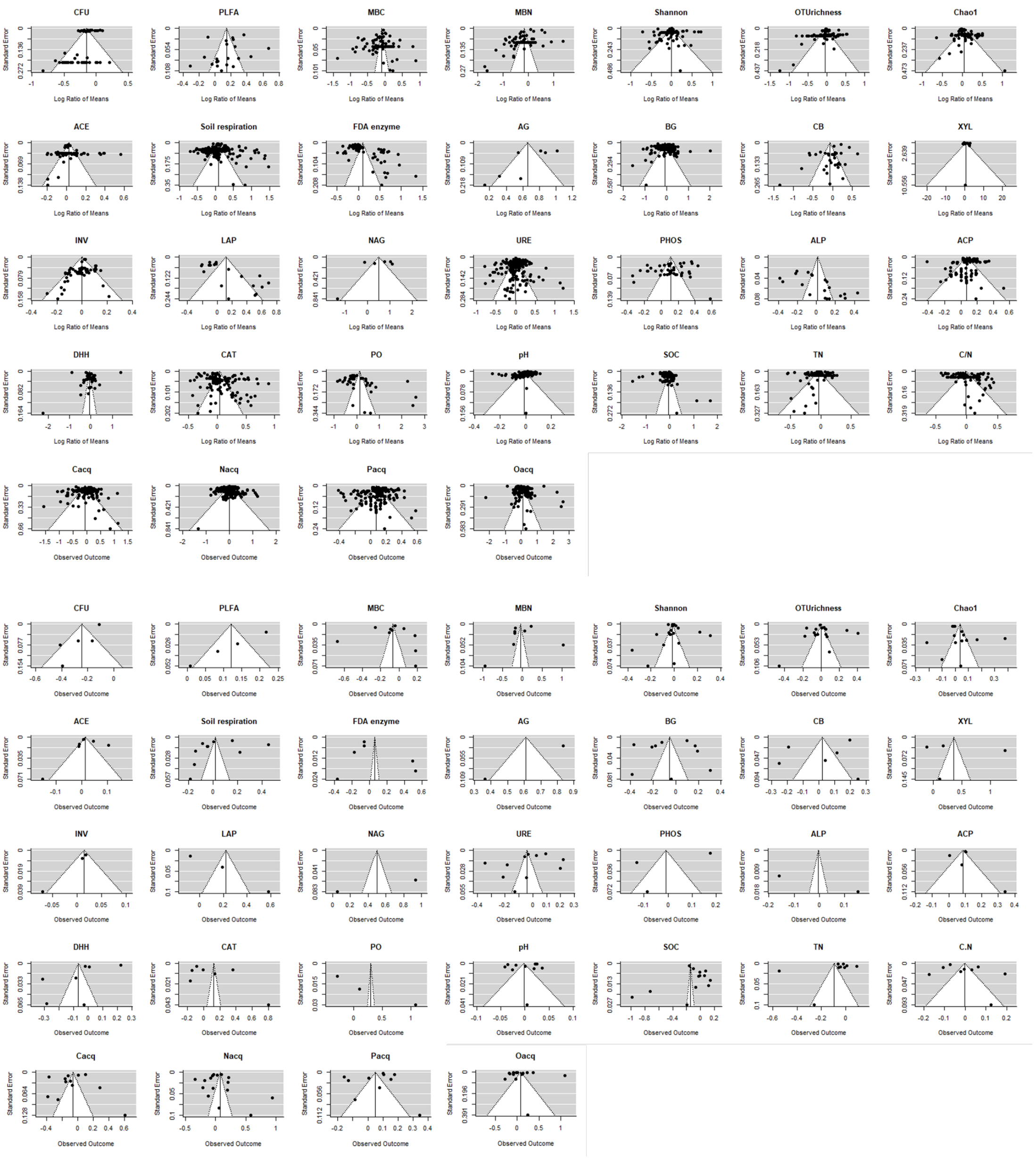

Fig. S2 Funnel plots for the effect of plastic residue on soil microbial parameters. The analyses using all cases as independent data were showed in upper panel and weighted mean response ratios aggregated within-study were showed in lower panel. The outer dashed lines indicate the triangular region within which 95% of studies are expected to lie in the absence of both biases and heterogeneity. The dashed vertical line indicates the summary effect estimate. There was no evidence of publication bias for all variables except Chao1 index, BG, INV, and LAP. The effects of plastic residue on BG, INV, and LAP were therefore adjusted using a trim-and-fill method. After the adjustment, no significant differences in neither direction nor significance of results between simulated and observed effect size, i.e., no missing studies need to be added to the current database. The results of fixed-effect model with the moderator of parameter of microbial richness indicate that using different metrics would not introduce much bias in richness analysis (see Fig. S3).

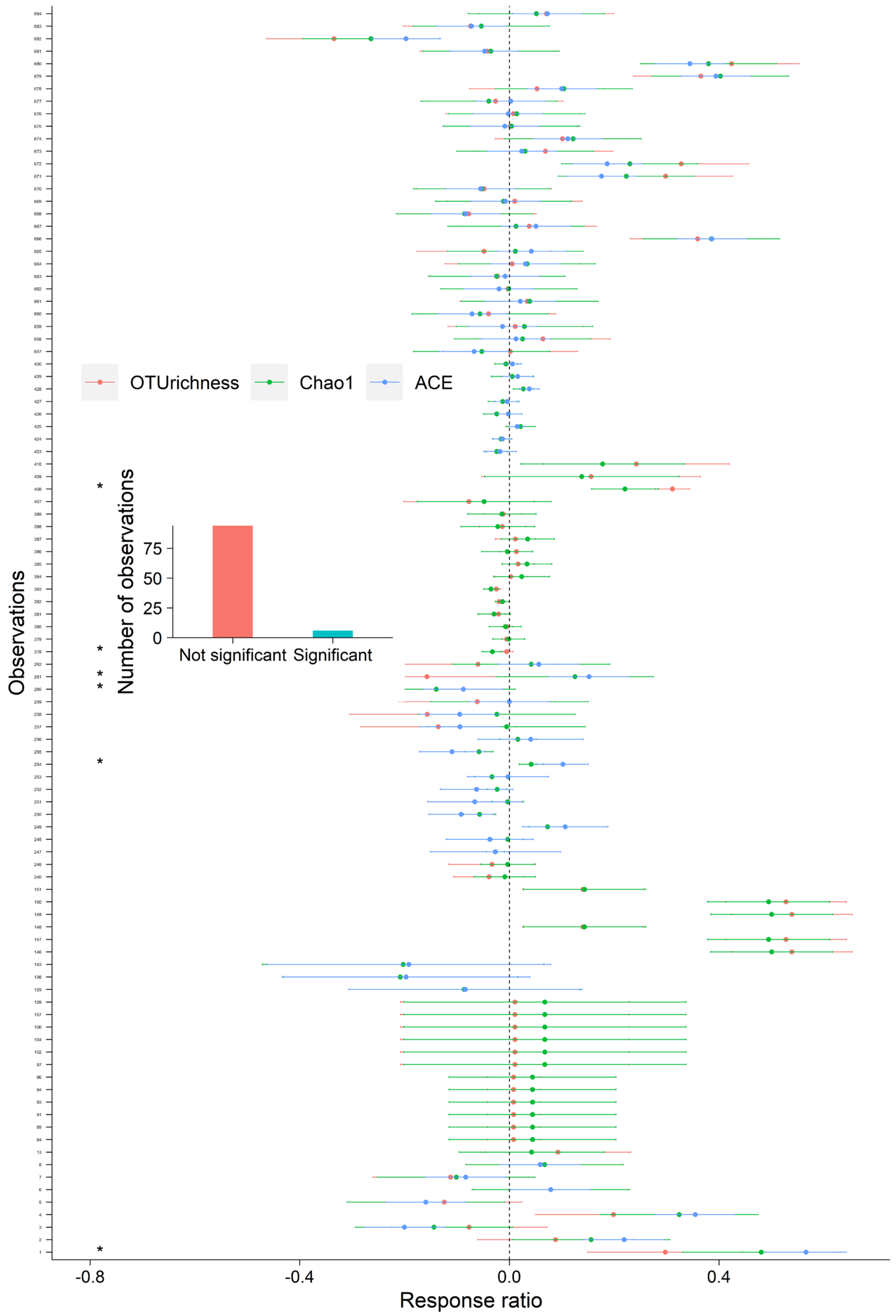

Fig. S3 The comparisons of the response ratios of different microbial richness parameters. The asterisks on the left indicate significant differences among the parameters based on omnibus test. The barplot in the middle-left panel shows the total number of significant and not significant observations.

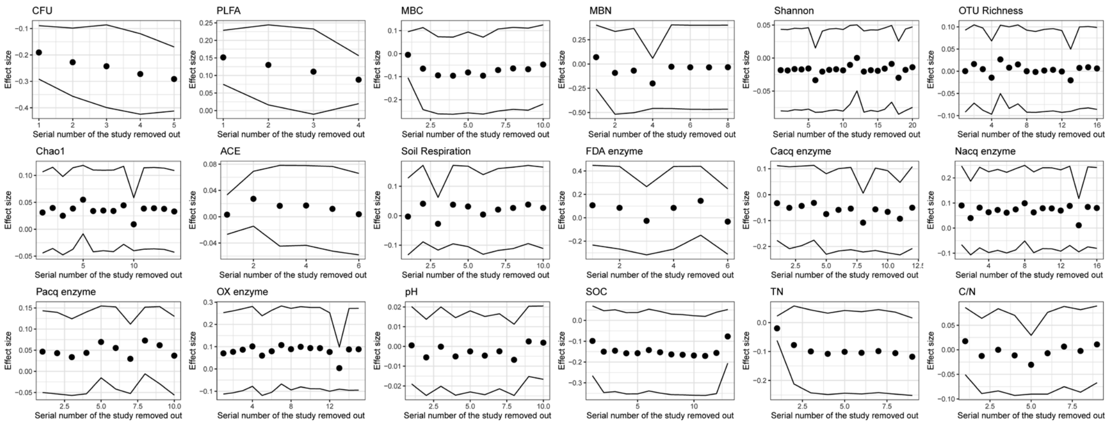

Fig. S4 Results of the Leave-one-out sensitivity tests conducted for the variables involved in the meta-analysis. of the effect size. The analyses showed no marked difference in results, suggesting that they were not driven by a single influential study. The black dots indicate the estimated effect sizes removing one study, and the black polyline lines above and below indicate the upper and lower confidence interval, respectively.

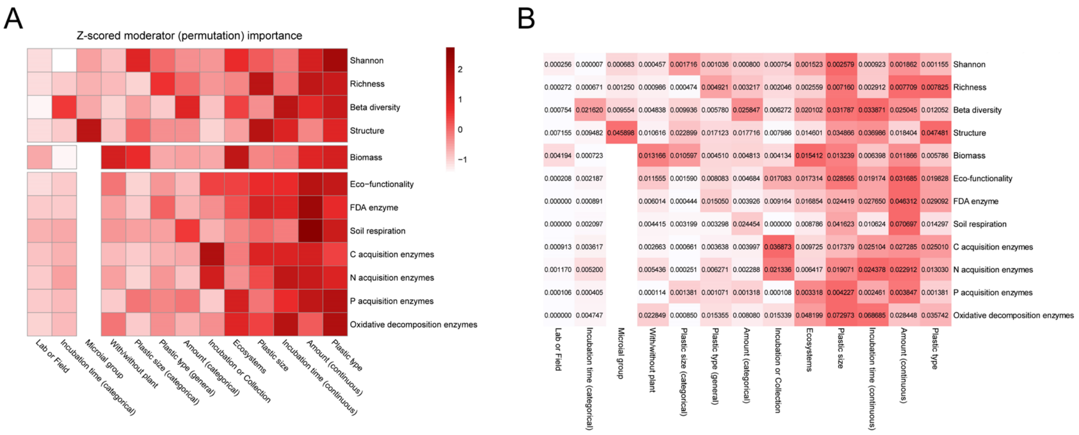

Fig. S5 The influence of selected explanatory variables on each soil microbial parameter based on the random forest approach consider the weight of samples. (A) The moderator importance to response variables were showed in red fine-grained heatmap, the values were Z-scored for the ease of visualizing. The detailed moderator importance values were showed in panel (B). The observations for the machine learning models in predicting variables from Shannon to Oxidative decomposition enzymes are: 153, 182, 72, 72, 97, 402, 72, 143, 184, 206, 130, and 203.

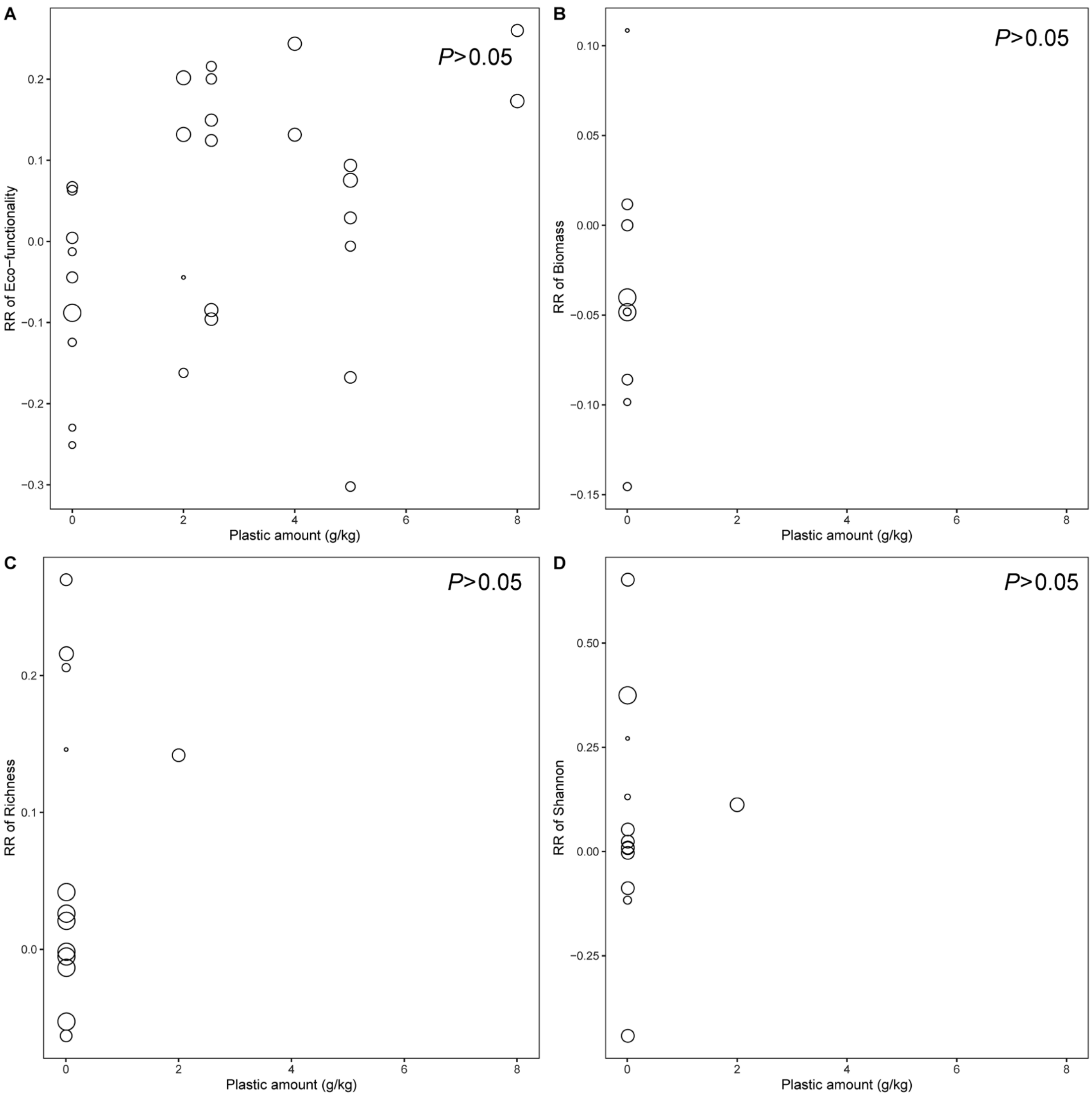

Fig. S6 Relationships between the response ratios of (A) soil microbial eco-functionality, (B) biomass, (C) richness, and (D) Shannon index and nanoplastic amount.

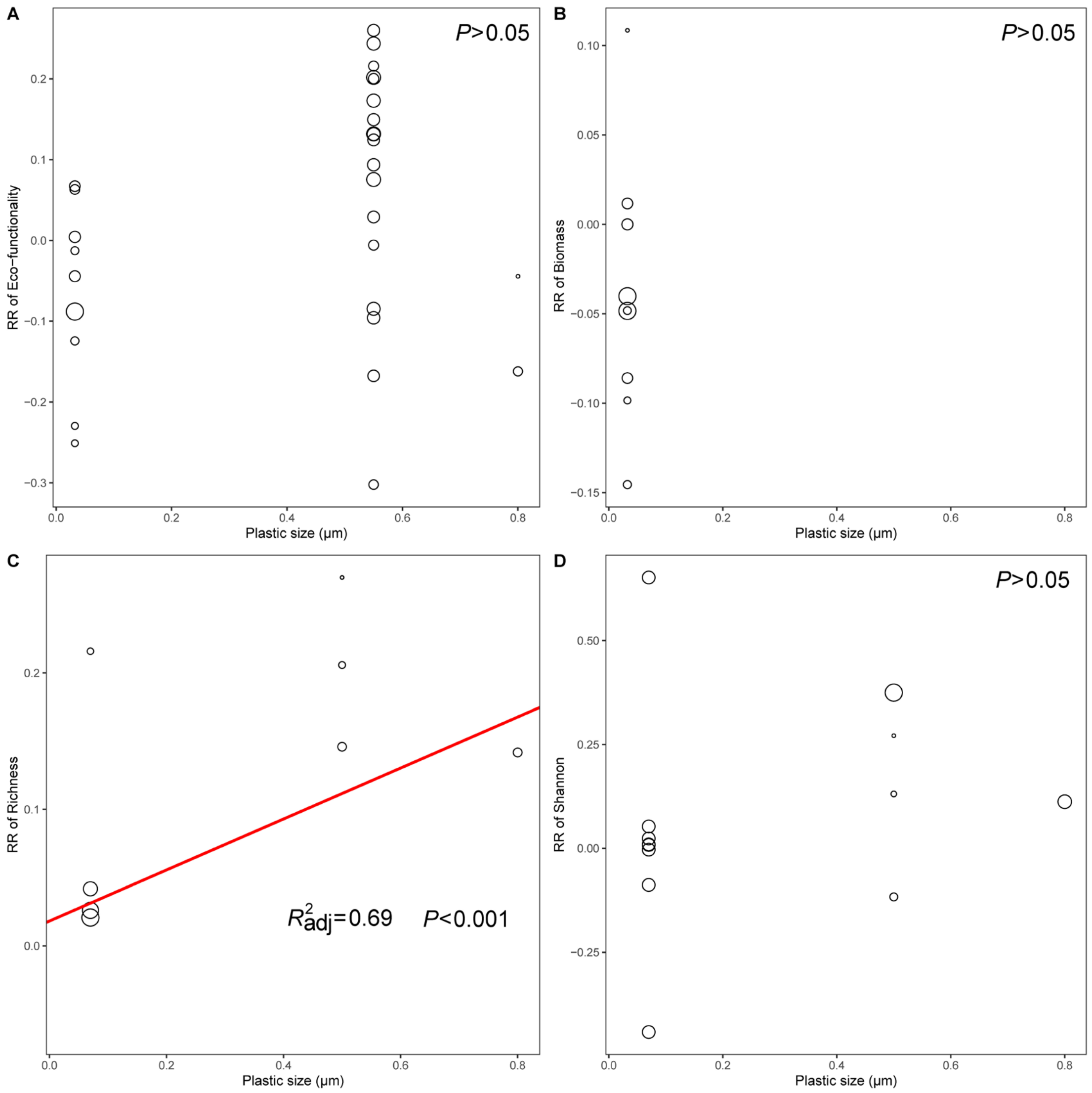

Fig. S7 Relationships between the response ratios of (A) soil microbial eco-functionality, (B) biomass, (C) richness, and (D) Shannon index and nanoplastic size.

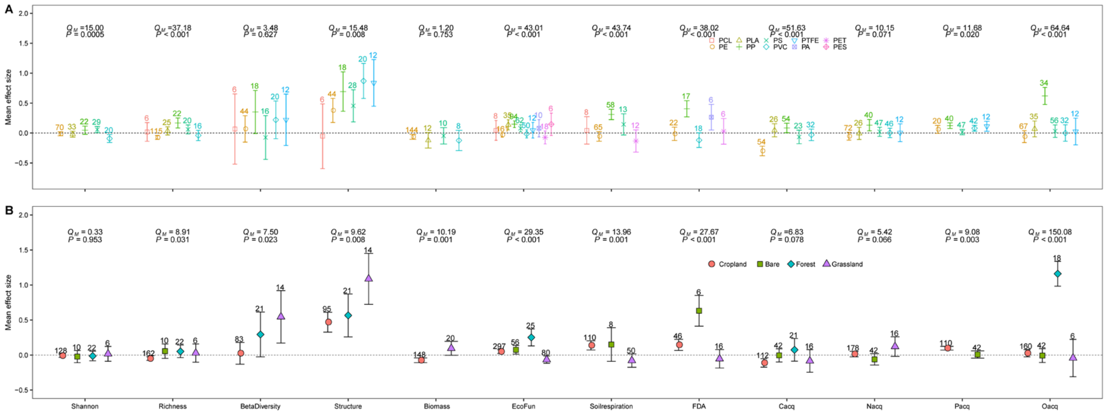

Fig. S8 Responses of the selected microbial community parameters to plastics across the different (A) plastic types and (B) biomes. The mean bar values expressed as the mean effect size of each variable with 95% confidence intervals (CIs). The sample size of each variable is given at the top of the CIs. The significance among categories within each subgroup was tested by the omnibus test (QM).

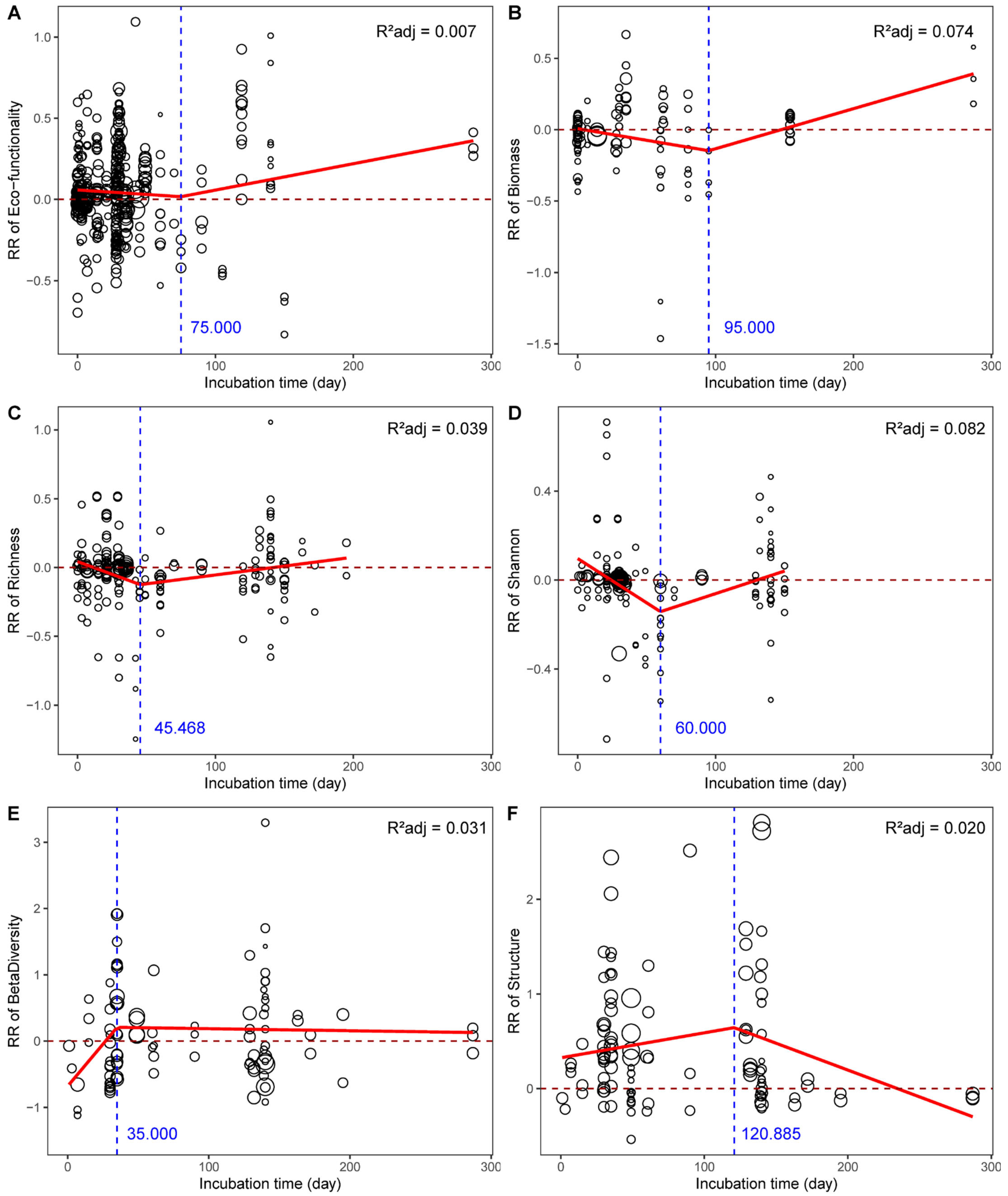

Fig. S9 Relationships between plastic incubation time and response ratio of soil microbial (A) functionality, (B) biomass, (C) richness, (D) Shannon index, (E) β-diversity, (F) community structure. The red solid line represents the fitted significant ($p < 0.05$) weighted linear model or broken-stick model, while the blue dashed line represents the infection point of broken-stick model. The sizes of dots were proportional to the weights of samples.

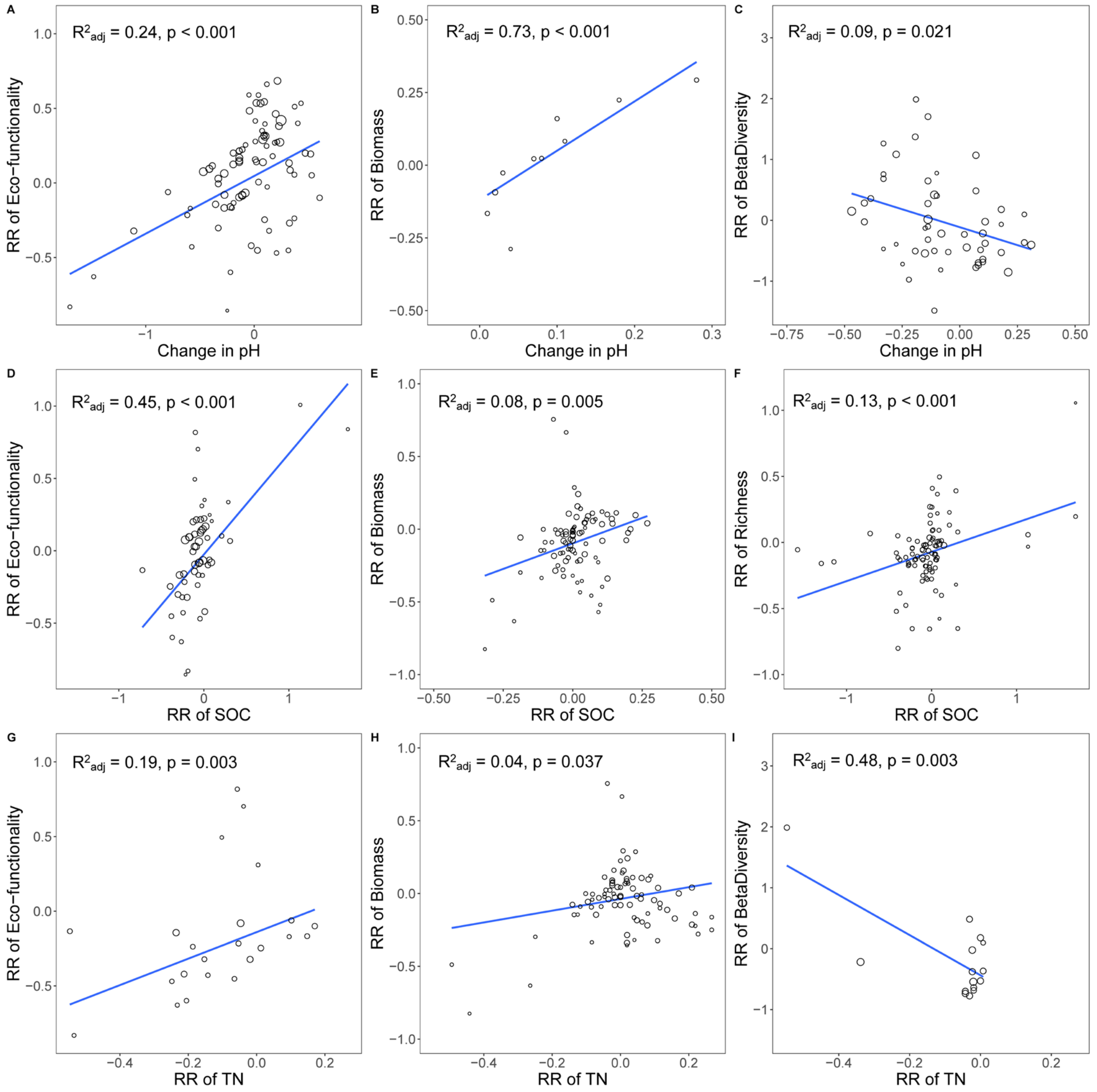

Fig. S10 Relationships between the response ratios of soil microbial (A) functionality, (B) biomass, and (C) β-diversity and change in pH induced by plastic residues. Relationships between the response ratios of soil microbial (D) functionality, (E) biomass, and (F) richness and response ratio of SOC induced by plastic residues. Relationships between the response ratios of soil microbial (G) functionality, (H) biomass, and (I) β-diversity and response ratio of TN induced by plastic residues. The sizes of dots were proportional to the weights of samples.

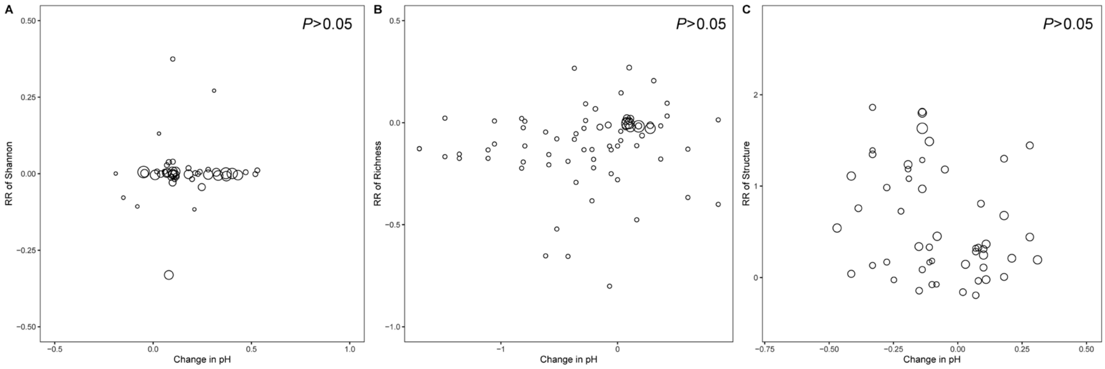

Fig. S11 Relationships between the response ratios of (A) soil microbial Shannon index, (B) richness, and (C) community structure and change in pH induced by plastic residues.

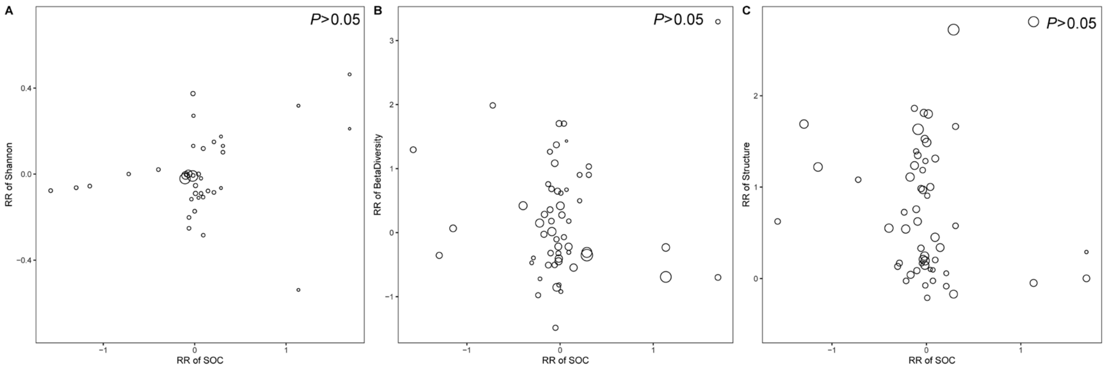

Fig. S12 Relationships between the response ratios of (A) soil microbial Shannon index, (B) beta diversity, and (C) community structure and change in SOC induced by plastic residues.

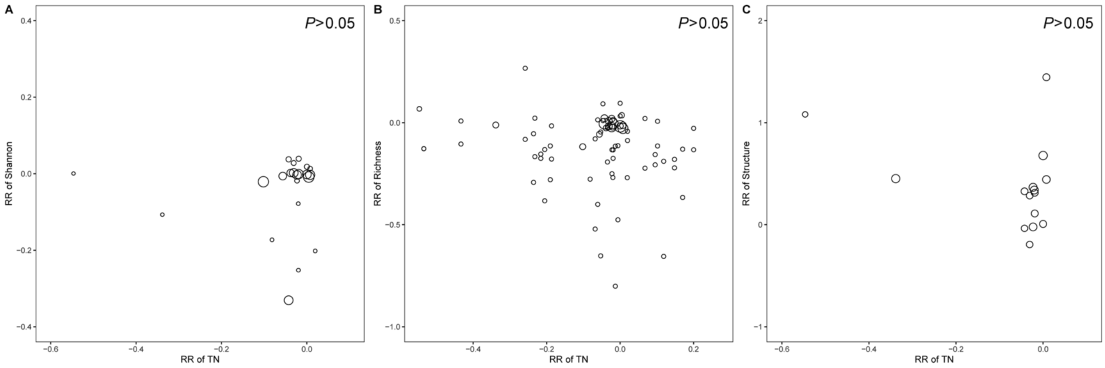

Fig. S13 Relationships between the response ratios of (A) soil microbial Shannon index, (B) richness, and (C) community structure and change in TN induced by plastic residues.

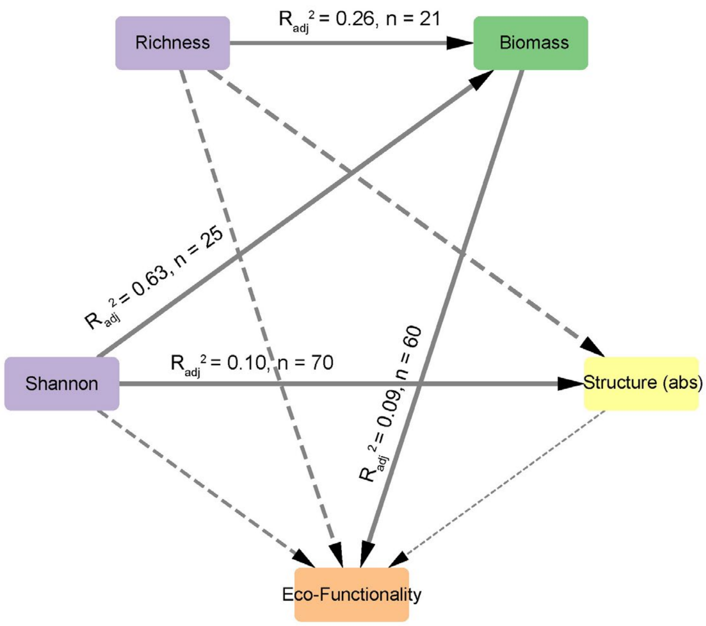

Fig. S14 The microbial biomass-diversity-functionality relationships among microbial community parameters based on their response ratios. The thickness of arrows represents the p value. Solid arrows represent significant ($p < 0.05$) relationships while dashed for insignificant relationships. The absolute value for microbial community structure is used since the community structure cannot be quantified as increasing or decreasing.

Supplementary Text S1. Data sources of this meta-analysis study.

Awet, T.T., Kohl, Y., Meier, F., Straskraba, S., Grun, A.L., Ruf, T., Jost, C., Drexel, R., Tunc, E., Emmerling, C., 2018. Effects of polystyrene nanoparticles on the microbiota and functional diversity of enzymes in soil. Environ Sci Eur 30. ***Country: Germany***

Blocker, L., Watson, C., Wichern, F., 2020. Living in the plastic age - Different short-term microbial response to microplastics addition to arable soils with contrasting soil organic matter content and farm management legacy. Environ Pollut 267. ***Country: Germany***

Chen, H.P., Wang, Y.H., Sun, X., Peng, Y.K., Xiao, L., 2020. Mixing effect of polylactic acid microplastic and straw residue on soil property and ecological function. Chemosphere 243. ***Country: China***

Dong, Y.M., Gao, M.L., Liu, X.W., Qiu, W.W., Song, Z.G., 2020. The mechanism of polystyrene microplastics to affect arsenic volatilization in arsenic-contaminated paddy soils. Journal of Hazardous Materials 398. ***Country: China***

Dong, Y.M., Gao, M.L., Qiu, W.W., Song, Z.G., 2021. Effect of microplastics and arsenic on nutrients and microorganisms in rice rhizosphere soil. Ecotox Environ Safe 211. ***Country: China***

Fei, Y.F., Huang, S.Y., Zhang, H.B., Tong, Y.Z., Wen, D.S., Xia, X.Y., Wang, H., Luo, Y.M., Barcelo, D., 2020. Response of soil enzyme activities and bacterial communities to the accumulation of microplastics in an acid cropped soil. Sci Total Environ 707. ***Country: China***

Gao, B., Yao, H.Y., Li, Y.Y., Zhu, Y.Z., 2021. Microplastic Addition Alters the Microbial Community Structure and Stimulates Soil Carbon Dioxide Emissions in Vegetable-Growing Soil. Environmental Toxicology and Chemistry 40, 352-365. ***Country: China***

Guo, Q.Q., Xiao, M.R., Ma, Y., Niu, H., Zhang, G.S., 2021. Polyester microfiber and natural organic matter impact microbial communities, carbon-degraded enzymes, and carbon accumulation in a clayey soil. J Hazard Mater 405, 124701. ***Country: China***

Hou, J., Xu, X., Yu, H., Xi, B., Tan, W., 2021. Comparing the long-term responses of soil microbial structures and diversities to polyethylene microplastics in different aggregate fractions. Environ Int 149, 106398. ***Country: China***

Hou, J.H., 2020. Effects of polyethylene microplastics on soil aggregate properties and microbial diversity. Master degree thesis, Lanzhou Jiaotong University. ***Country: China***

Huang, L., 2017. Impact of polyethylene film residues on crop fields soil quality and soil microbial diversity. Master degree thesis, Lanzhou University. ***Country: China***

Huang, Y., Zhao, Y.R., Wang, J., Zhang, M.J., Jia, W.Q., Qin, X., 2019. LDPE microplastic films alter microbial community composition and enzymatic activities in soil. Environ Pollut 254. ***Country: China***

Koskei, K., Munyasya, A.N., Wang, Y.B., Zhao, Z.Y., Zhou, R., Indoshi, S.N., Wang, W., Cheruiyot, W.K., Mburu, D.M., Nyende, A.B., Xiong, Y.C., 2021. Effects of increased plastic film residues on soil properties and crop productivity in agro-ecosystem. Journal of Hazardous Materials 414. ***Country: China***

Lin, D., Yang, G., Dou, P., Qian, S., Zhao, L., Yang, Y., Fanin, N., 2020. Microplastics negatively affect soil fauna but stimulate microbial activity: insights from a field-based microplastic addition experiment. Proc Biol Sci 287, 20201268. ***Country: China***

Liu, H., Yang, X., Liu, G., Liang, C., Xue, S., Chen, H., Ritsema, C.J., Geissen, V., 2017. Response of soil dissolved organic matter to microplastic addition in Chinese loess soil. Chemosphere 185, 907-917. ***Country: China***

Liu, Y., Huang, Q., Hu, W., Qin, J.M., Zheng, Y.R., Wang, J.F., Wang, Q.Q., Xu, Y.X., Guo, G.M., Hu, S., Xu, L., 2021. Effects of plastic mulch film residues on soil-microbe-plant systems under different soil pH conditions. Chemosphere 267. ***Country: China***

Lozano, Y.M., Aguilar-Trigueros, C.A., Onandia, G., Maaß, S., Zhao, T., Rillig, M.C., 2021a. Effects of microplastics and drought on soil ecosystem functions and multifunctionality. Journal of Applied Ecology 58, 988-996. ***Country: Germany***

Lozano, Y.M., Lehnert, T., Linck, L.T., Lehmann, A., Rillig, M.C., 2021b. Microplastic Shape, Polymer Type, and Concentration Affect Soil Properties and Plant Biomass. Front Plant Sci 12, 616645. ***Country: Germany***

Machado, A.A.D., Lau, C.W., Kloas, W., Bergmann, J., Bacheher, J.B., Faltin, E., Becker, R., Gorlich, A.S., Rillig, M.C., 2019. Microplastics Can Change Soil Properties and Affect Plant Performance. Environ Sci Technol 53, 6044-6052. ***Country: Germany***

Machado, A.A.D., Lau, C.W., Till, J., Kloas, W., Lehmann, A., Becker, R., Rillig, M.C., 2018. Impacts of Microplastics on the Soil Biophysical Environment. Environ Sci Technol 52, 9656-9665. ***Country: Germany***

Moore-Kucera, J., Cox, S.B., Peyron, M., Bailes, G., Kinloch, K., Karich, K., Miles, C., Inglis, D.A., Brodhagen, M., 2014. Native soil fungi associated with compostable plastics in three contrasting agricultural settings. Appl Microbiol Biot 98, 6467-6485. ***Country: USA***

Ng, E.L., Lin, S.Y., Dungan, A.M., Colwell, J.M., Ede, S., Lwanga, E.H., Meng, K., Geissen, V., Blackall, L.L., Chen, D.L., 2021. Microplastic pollution alters forest soil microbiome. Journal of Hazardous Materials 409. ***Country: Australia***

Nong, M.M., 2013. The Effects of LMWPE on soil microbial activities and the screening of LMWPE degrading microorganism. Master degree thesis, Sichuan Normal University. ***Country: USA***

Qi, Y., Ossowicki, A., Yang, X., Huerta Lwanga, E., Dini-Andreote, F., Geissen, V., Garbeva, P., 2020. Effects of plastic mulch film residues on wheat rhizosphere and soil properties. J Hazard Mater 387, 121711. ***Country: Netherlands***

Qian, H.F., Zhang, M., Liu, G.F., Lu, T., Qu, Q., Du, B.B., Pan, X.L., 2018. Effects of Soil Residual Plastic Film on Soil Microbial Community Structure and Fertility. Water Air Soil Poll 229. ***Country: China***

Ren, X., Tang, J., Liu, X., Liu, Q., 2020. Effects of microplastics on greenhouse gas emissions and the microbial community in fertilized soil. Environ Pollut 256, 113347. ***Country: China***

Ren, X.W., Tang, J.C., Wang, L., Liu, Q.L., 2021. Microplastics in soil-plant system: effects of nano/microplastics on plant photosynthesis, rhizosphere microbes and soil properties in soil with different residues. Plant Soil 462, 561-576. ***Country: China***

Rong, L., Zhao, L., Zhao, L., Cheng, Z., Yao, Y., Yuan, C., Wang, L., Sun, H., 2021. LDPE microplastics affect soil microbial communities and nitrogen cycling. Sci Total Environ 773, 145640. ***Country: China***

Song, C., 2014. Impact of polyethylene film residues on crop yields, soil quality and soil microbial community composition. PhD degree thesis, Lanzhou University. ***Country: China***

Song, S.J., Zhao, X.G., Nie, W.J., Zhang, Y., Li, Y., Zhao, X., 2016. Effect of Plastic Film Residue on Soil Microorganism Based on Pot Experiment. Northern Horticulture 4, 172-178. ***Country: China***

Wang, J., Liu, X., Dai, Y., Ren, J., Li, Y., Wang, X., Zhang, P., Peng, C., 2020. Effects of co-loading of polyethylene microplastics and ciprofloxacin on the antibiotic degradation efficiency and microbial community structure in soil. Sci Total Environ 741, 140463. ***Country: China***

Wang, J., Lv, S., Zhang, M., Chen, G., Zhu, T., Zhang, S., Teng, Y., Christie, P., Luo, Y., 2016. Effects of plastic film residues on occurrence of phthalates and microbial activity in soils. Chemosphere 151, 171-177. ***Country: China***

Wei, H.D., Lun, Z.L., Guo, F., 2008. Effects of mulch film residues on soil properties. Ecology and Environment 17, 1853-1856. ***Country: China***

Wiedner, K., Polifka, S., 2020. Effects of microplastic and microglass particles on soil microbial community structure in an arable soil (Chernozem). Soil-Germany 6, 315-324. ***Country: Germany***

Wijesekara, H., Bolan, N.S., Bradney, L., Obadamudalige, N., Seshadri, B., Kunhikrishnan, A., Dharmarajan, R., Ok, Y.S., Rinklebe, J., Kirkham, M.B., Vithanage, M., 2018. Trace element dynamics of biosolids-derived microbeads. Chemosphere 199, 331-339. ***Country: Australia***

Xu, M.L., Du, W.C., Ai, F.X., Xu, F., Zhu, J.G., Yin, Y., Ji, R., Guo, H.Y., 2021. Polystyrene microplastics alleviate the effects of sulfamethazine on soil microbial communities at different CO2 concentrations. Journal of Hazardous Materials 413. ***Country: China***

Xu, Z., Qian, X., Wang, C., Zhang, C., Tang, T., Zhao, X., Li, L., 2020. Environmentally relevant concentrations of microplastic exhibits negligible impacts on thiacloprid dissipation and enzyme activity in soil. Environ Res 189, 109892. ***Country: China***

Yan, Y.Y., Chen, Z.H., Zhu, F.X., Zhu, C.Y., Wang, C., Gu, C., 2021. Effect of Polyvinyl Chloride Microplastics on Bacterial Community and Nutrient Status in Two Agricultural Soils (vol 53, pg 613, 2020). B Environ Contam Tox 106, 236-236. ***Country: China***

Yang, W.W., 2020. The influence of microplastics on biological effects of nanoscale ZnO in soil. Master degree thesis, Lanzhou Jiaotong University. Qingdao University Of Science & Technology. ***Country: China***

Yang, X.M., Bento, C.P.M., Chen, H., Zhang, H.M., Xue, S., Lwanga, E.H., Zomer, P., Ritsema, C.J., Geissen, V., 2018. Influence of microplastic addition on glyphosate decay and soil microbial activities in Chinese loess soil. Environ Pollut 242, 338-347. ***Country: China***

Yi, M.L., Zhou, S.H., Zhang, L.L., Ding, S.Y., 2021. The effects of three different microplastics on enzyme activities and microbial communities in soil. Water Environ Res 93, 24-32. ***Country: China***

Zang, H.D., Zhou, J., Marshall, M.R., Chadwick, D.R., Wen, Y., Jones, D.L., 2020. Microplastics in the agroecosystem: Are they an emerging threat to the plant-soil system? Soil Biol Biochem 148. ***Country: UK***

Zhang, D., Liu, H.B., Ma, Z.M., Tang, W.X., Wei, T., Yang, D.H., Li, J.G., Wang, H.Y., 2017. Effect of Residual Plastic Film on Soil Nutrient Contents and Microbial Characteristics in the Farmland. Scientia Agricultura Sinica 50, 310-319. ***Country: China***

Zhang, J., Chen, J., Jia, R.M., Dun, Z.H., Wang, B.T., Hu, X.P., Wang, Y., 2018. Selection and evaluation of microorganisms for biodegradation of agricultural plastic film. 3 Biotech 8. ***Country: China***

Zhang, M., Jia, H., Weng, Y.X., Li, C.T., 2020. Effects of polylactide/polybutylene adipate-co-terephthalate on bacterial community structure of soil and isolation of degrading bacteria. Microbiology China 47, 420-430. ***Country: China***

Zhang, Y., Liu, N., Qu, T., Sun, T., Li, D.P., Wang, Z.X., Zhao, X.N., Jia, H.T., 2019. Effects of Residues of Different Plastic Films on Soil Respiration Rate. Journal of Xinjiang Agricultural University 42, 210-215. ***Country: China***

Zhao, X., 2018. Effects of Accumulation of Plastic Film on Soil Microbial Activity. Industrial Safety and Environmental Protection 44, 94-96. ***Country: China***

Zhao, X., Luo, L., 2018. Analysis of the influence of plastic film residue on soil biological environment: taking cucumber mulching film residue soil as an example. Rural science and technology 6, 100-101. ***Country: China***

References:

Chen J, Luo Y, van Groenigen KJ, Hungate BA, Cao J, Zhou X, Wang RW (2018) A keystone microbial enzyme for nitrogen control of soil carbon storage. Sci Adv 4: eaaq1689. doi: 10.1126/sciadv.aaq1689.

de Souza Machado AA, Kloas W, Zarfl C, Hempel S, Rillig MC (2018) Microplastics as an emerging threat to terrestrial ecosystems. Global Change Biol 24: 1405-1416. doi: 10.1111/gcb.14020.

Duval S, Tweedie R (2000) Trim and fill: A simple funnel-plot-based method of testing and adjusting for publication bias in meta-analysis. Biometrics 56: 455-463. doi: DOI 10.1111/j.0006-341X.2000.00455.x.

Lozano YM, Aguilar-Trigueros CA, Onandia G, Maaß S, Zhao T, Rillig MC (2021) Effects of microplastics and drought on soil ecosystem functions and multifunctionality. Journal of Applied Ecology 58: 988-996. doi: https://doi.org/10.1111/1365-2664.13839.

Lu XF, Hou EQ, Guo JY, Gilliam FS, Li JL, Tang SB, Kuang YW (2021) Nitrogen addition stimulates soil aggregation and enhances carbon storage in terrestrial ecosystems of China: A meta-analysis. Global Change Biol 27: 2780-2792. doi: 10.1111/gcb.15604.

Nessel MP, Konnovitch T, Romero GQ, Gonzalez AL (2021) Nitrogen and phosphorus enrichment cause declines in invertebrate populations: a global meta-analysis. Biol Rev 96: 2617-2637. doi: 10.1111/brv.12771.

Rosenberg MS (2005) The file-drawer problem revisited: A general weighted method for calculating fail-safe numbers in meta-analysis. Evolution 59: 464-468. doi: Doi 10.1554/04-602.

Song C, Peacor SD, Osenberg CW, Bence JR (2020) An assessment of statistical methods for nonindependent data in ecological meta-analyses. Ecology 101. doi: 10.1002/ecy.3184.

Wang J, Peng C, Li HY, Zhang PP, Liu XH (2021) The impact of microplastic-microbe interactions on animal health and biogeochemical cycles: A mini-review. Sci Total Environ 773. doi: ARTN 145697

10.1016/j.scitotenv.2021.145697.

Zhou Z, Wang C, Luo Y (2020) Meta-analysis of the impacts of global change factors on soil microbial diversity and functionality. Nat Commun 11: 3072. doi: 10.1038/s41467-020-16881-7.
